# Secretion of miRNA-326-3p by senescent adipose exacerbates myocardial metabolism in diabetic mice

**DOI:** 10.1186/s12967-022-03484-7

**Published:** 2022-06-21

**Authors:** Hao Lin, Xiaonan Chen, Jianan Pan, Jiahan Ke, Alian Zhang, Yangyang Liu, Changqian Wang, Alex Chia Yu Chang, Jun Gu

**Affiliations:** 1 Department of Cardiology, Shanghai Ninth People’s Hospital, Shanghai JiaoTong University School of Medicine, Shanghai, China; 2 Shanghai Institute of Precision Medicine, Shanghai Ninth People’s Hospital, Shanghai JiaoTong University School of Medicine, Shanghai, China

**Keywords:** Diabetic cardiomyopathy, Cardiomyocytes, Adipose tissue, Senescence, Extracellular vesicles

## Abstract

**Background:**

Adipose tissue homeostasis is at the heart of many metabolic syndromes such as diabetes. Previously it has been demonstrated that adipose tissues from diabetic patients are senescent but whether this contributes to diabetic cardiomyopathy (DCM) remains to be elucidated.

**Methods:**

The streptozotocin (STZ) type 1 diabetic mice were established as animal model, and adult mouse ventricular myocytes (AMVMs) isolated by langendorff perfusion as well as neonatal mouse ventricular myocytes (NMVMs) were used as cell models. Senescent associated β galactosidase (SA-β-gal) staining and RT-qPCR were used to identify the presence of adipose senescence in diabetic adipose tissue. Senescent adipose were removed either by surgery or by senolytic treatment. Large extracellular vesicles (LEVs) derived from adipose tissue and circulation were separated by ultracentrifugation. Cardiac systolic and diastolic function was evaluated through cardiac ultrasound. Cardiomyocytes contraction function was evaluated by the Ionoptix HTS system and live cell imaging, mitochondrial morphology and functions were evaluated by transmission electron microscope, live cell fluorescent probe and seahorse analysis. RNA-seq for AMVMs and miRNA-seq for LEVs were performed, and bioinformatic analysis combined with RT-qPCR and Western blot were used to elucidate underlying mechanism that senescent adipose derives LEVs exacerbates myocardial metabolism.

**Results:**

SA-β-gal staining and RT-qPCR identified the presence of adipose tissue senescence in STZ mice. Through surgical as well as pharmacological means we show that senescent adipose tissue participates in the pathogenesis of DCM in STZ mice by exacerbates myocardial metabolism through secretion of LEVs. Specifically, expression of miRNA-326-3p was up-regulated in LEVs isolated from senescent adipose tissue, circulation, and cardiomyocytes of STZ mice. Up-regulation of miRNA-326-3p coincided with myocardial transcriptomic changes in metabolism. Functionally, we demonstrate that miRNA-326-3p inhibited the expression of Rictor and resulted in impaired mitochondrial and contractile function in cardiomyocytes.

**Conclusion:**

We demonstrate for the first time that senescent adipose derived LEVs exacerbates myocardial metabolism through up-regulated miRNA-326-3p which inhibits Rictor in cardiomyocytes. Furthermore, reducing senescence burden in adipose tissue is capable of relieving myocardial metabolism disorder in diabetes mellitus.

**Supplementary Information:**

The online version contains supplementary material available at 10.1186/s12967-022-03484-7.

## Background

Cardiovascular complications of diabetes are one of the leading causes of death in diabetic patients [1]. Diabetic cardiomyopathy is defined as structural and functional abnormalities of the myocardium in the absence of other risk factors such as hypertension, coronary artery disease, and valvular disease [2] and often results in metabolic disorder in cardiomyocytes [3]. Fat mass correlates extremely well with risk of cardiovascular complications in diabetic patients: increase in visceral fat correlates with an increase in cardiovascular risk for both type 1 and type 2 diabetes [4–6]. As the largest metabolic organ, the secretory phenotype of adipose tissue plays an important role in the regulation of the metabolism of removal organs at both physiological and pathological states, especially cardiomyocytes [7] given its high energy expenditure. Extracellular vehicles (EVs) are membrane-structured vectors that carry proteins, lipids, and/or non-coding RNAs, which contribute to inter-organ and inter-cellular signaling [8]. Depending on the particle diameter, extracellular vesicles can be classified as small EVs (SEVs, 30–100 nm) and large EVs (LEVs, 100 nm–1 µm) [9]. In adipose tissue, SEVs and LEVs are secreted in comparable amounts but have distinctly different content and composition properties [10]. In dysfunctional adipose, secretion of SEVs as well as LEVs are increased [11, 12]. Studies on adipose derived EVs have largely focused on SEVs [13, 14], yet the role of LEVs remains largely unknown.

Premature senescence is an important pathologic change often induced by cellular stress. Premature senescence has been shown to cause, drive, and potentially serve as therapeutic targets for combating aging associated diseases such as neurological disorders, diabetes, cardiovascular diseases, and cancer [15, 16]. Oxidative stress and hyperglycemia are two major factors that can cause premature senescence [17, 18]. When stress-induced DNA damage occurs, DNA-damage response pathway regulators p53 and p16 are activated [19, 20] and can result in adipose senescence [21]. Senescent cells are bad neighbors that are capable of spreading senescence, locally or distally, through paracrine factors termed senescence-associated secretory phenotype (SASP) [22, 23]. Interestingly, adipose tissue from diabetic patients as well as mice have been shown to exhibit senescent markers [24, 25] but whether senescent adipose participates in the pathogenesis of diabetes remains to be elucidated.

Using the STZ murine model, here we show that LEVs derived from senescent adipose are responsible for inducing diastolic dysfunction in the heart. We identify and demonstrate that miR-326-3p, detected in senescent adipose derived LEVs, acts distally in the heart. Mechanistically, miR-326-3p downregulates Rictor and results in mitochondrial respiration dysfunction in cardiomyocytes. Finally, we demonstrate that senescent adipose removal blocks the secretion of miR-326-3p containing LEVs and prevents the onset of cardiac diastolic dysfunction. Our results provide new evidence for diabetic cardiomyopathy progression and give support for future detection and therapeutic targeting for diabetic cardiomyopathies.

## Methods

### Experimental animals

The study followed the guidelines for the care and use of laboratory animals as outlined by the "Guides for the Care and Use of Laboratory Animals". The Animal Experiment Ethics Committee of Shanghai Ninth People’s Hospital approved all experiments [SH9H-2019-A416-1]. Wild type C57BL/6 J adult mice were obtained from GemPharmatech Co., Ltd. Animals were under a light/dark cycle of 12:12 h (lights on at 6:00) at 22 ± 2 °C. Mice got a standard chow diet (3.236 kcal/g; 13.5% of calories were lipids; 25.2% were proteins; 61.3% were carbohydrates; Xietong) and water was available ad libitum. Diabetes mellitus was induced in 8-weeks-old C57BL/6 J mice, the mice were injected intraperitoneally (i.p.) with Streptozocin (STZ; Sigma-Aldrich) after fasting for 12 h. STZ was dissolved in citrate buffer (Beyotime) and injected within 30 min of dissolution. Two weeks after STZ injection, blood glucose was measured with tail vein sampling by Accu-Check (Roche). Epididymis adipose tissue removal (EATr) was performed as previous described [26], briefly, 6 h after STZ injection, 3% isoflurane (RWD Life Science) was inhaled to anesthetize the mice and a 1-cm midline abdominal incision was made. The epididymal adipose tissue were dissected using an operating scalpel and removed from the peritoneal cavity without damaging the testicular blood supply. Sham-operation was performed the same way, but without removing the adipose tissue. For senolytic treatment, dasatinib (5 mg/kg; MCE) and quercetin (50 mg/kg; Sigma- Aldrich) or vehicle (4% DMSO, 40% PEG-400, and 54% Saline) were given through oral gavage for 3 days every 2 weeks.

### Cardiomyocytes preparation

Adult mouse ventricular myocytes (AMVMs) were isolated using a Langendorff perfusion system. The mice were anesthetized with 5% isoflurane and sacrificed by cervical dislocation. Hearts were isolated and immediately digested through perfusion with enzyme cardiomyocyte isolation buffer (120 mM NaCl, 5.4 mM KCl, 0.5 mM MgSO_4_, 0.33 mM NaH_2_PO_4,_ 25 mM NaHCO_3_, and 22 mM glucose, 25 mM HEPES, 10 mM BDM, 30 mM taurine,1 mg/ml Type II Collagenase, 0.6 mg/ml Type IV Collagenase; CIB) for 15 min at 37 °C. The left ventricle was minced and dissociated in Minimum Essential Medium Eagle (MEM; Sigma-Aldrich) supplemented with 10% bovine serum albumin (BSA; Sigma-Aldrich). After filtration, AMVMs were collected under 500 rpm centrifugation. Calcium was reintroduced in 4 stages, ranging from 0 to 900 µM.

Neonatal mouse ventricular myocytes (NMVMs) were isolated from one-day old C57BL/6 J pups as previously described [27]. More than 95% of cardiomyocytes are typically present in cell preparations. The NMVMs were cultured in Dulbecco's modified eagle medium (DMEM; Thermo Fisher) with 10% fatal bovine serum (FBS; Thermo Fisher).

### Adipose-tissue culture

Epididymis adipose tissue (EAT) were obtained freshly. The EAT were then washed with PBS containing 1% Penicillin–Streptomycin (Thermo Fisher) and cut into small pieces. DMEM/F12 (Procell) were used as tissue culture medium containing 1 mM sodium pyruvate, 2 mM glutamine, MEM vitamins, MEM nonessential amino acids as previously described [28]. 48 h doxorubicin (0.2 mM, MCE) treatment were used to induce senescence. For senolytic treatment, quercetin (20 µM) and dasatinib (1 µM) were added into culture medium for 48 h.

### LEVs purification and identification

Murine sera were obtained within 2 h by centrifuging blood collected from mouse twice at 1000*g* for 5 min each. For the separation of whole cells, cellular debris and aggregates, murine sera samples were centrifuged at 1500*g* for 15 min followed by centrifugation at 20,000*g* for 40 min, respectively. After that, the platelet-deprived samples were immediately stored at − 80 °C. Adipose tissue supernatant was collected and centrifuged at 1500*g* for 15 min to remove cell debris. To pellet the LEVs, the supernatant was centrifuged again at 20,000*g* for 40 min. Pelleted LEVs were suspended in phosphate buffered saline (PBS, Thermo Fisher), ultra-centrifuged again at 20,000*g* for 40 min and resuspended in PBS for further study. LEVs were used in all experiments at a concentration of 2000 particles/μl in accordance to previous study [29]. The morphology was examined by transmission electron microscope (Thermo Fisher) using negative staining method, and the particle sizes and numbers were quantified by Nano Sight analysis (Malvern Instruments).

### LEVs trafficking detection

The PKH67 Green Fluorescent Cell Linker Mini Kit (Sigma Aldrich) was used to label LEVs with fluorescent dye in order to monitor LEV trafficking in vitro. The PKH67-labeled LEVs were cultured with NMVMs for 4 h following to the manufacturer's instructions. The cells were then fixed with 4% paraformaldehyde (Servicebio) for 10 min at room temperature. After washed twice with PBS, the cells were stained with blocking buffer with 1 µg/ml DAPI (4′,6-diamidino-2′-phenylindole, dihydrochloride; Sigma-Aldrich) for 5 min at room temperature. For live cell imaging, the NMVMs were incubated with the PKH67-labeled LEVs for 4 h, followed by Hoechst 33342 (Beyotime) staining for 15 min. Next, the cells were washed twice with PBS, and medium were changed to DMEM with 10% FBS (complete culture media).

### Transfection of miRNA mimics and inhibitors into NMVMs

The miR-326-3p mimic (Ruibio), miR-326-3p inhibitor (Ruibio) and their corresponding negative controls (NC; Ruibio) were transfected using Lipofectamine 3000 (Thermo Fisher) following manufacturer’s protocol. After 6 h, the media were changed to complete culture media. 24 h after the NMVMs transfected with miRNA inhibitors, the cells were treated with LEVs or same volume of culture media for another 36 h.

### Echocardiography

Transthoracic echocardiography was performed using a Visual Sonics Vevo 3100 system equipped with MS400 transducer (FUJIFILM Visual Sonics). Long axis M-mode scans at midventricular level were used to measure left ventricular systolic function. To assess diastolic function, apical 4-chamber views were used in anesthetized mice testing pulsed-wave and tissue Doppler at the level of mitral valve. The heart rates were maintained at 420–480 beats per min for systolic function assessment and 320–380 beats per min for diastolic function assessment.

### Cardiomyocyte contractility and calcium handle assay

IonOptix LLC's contractility/photometry system (IonOptix) was used to measure the contraction and calcium transients of isolated AMVMs. Briefly, isolated AMVMs were seeded onto glass bottom dishes pretreated by laminin (Sigma-Aldrich). After incubating in complete culture media with Fura-2 calcium Indicator (Invitrogen) for 30 min at cell incubator (37 °C, 5% CO_2_), a field stimulator was used to electrically stimulate AMVMs at 1 Hz, and changes of sarcomeric length and calcium transients was measured.

For NMVMs contractility assay, isolated NMVMs were seeded on 96-well plate (Perkin Elmer) 7 days prior to assay. The spontaneous contraction video of NMVMs were shooting by microscopy (Olympus). NMVMs were maintained at 37 °C with 5% CO_2_ to maintain physiological conditions. Contraction speed and contraction frequency were calculated using an established method in Matlab [30, 31].

### Mitochondrial respiration measurement

The cardiomyocytes mitochondrial respiration was measured using seahorse XF Cell Mito Stress Test Kit (Agilent) and performed by Seahorse XFe96 (Agilent). Briefly, Cardiomyocytes were seeded onto XFe96 Microplates (Agilent) 7 days (NMVMs) or 1 h (AMVMs) prior to experiments. The completed culture media were changed to Seahorse XF DMEM (Agilent) supplemented with 5 µM glucose, 1 µM pyruvate and 10 µM glutamine. After 1 h incubation at 37 °C CO_2_-free incubator, the Microplates were loaded into Seahorse XFe96 to assay mitochondrial respiration as manufacturer’s instructions.

### Mitochondrial membrane potential and mitochondrial superoxide assay

Mitochondrial membrane potential was measured using a Tetramethylrhodamine, methyl ester (TMRM) dye (Invitrogen). Mitochondrial superoxide levels were measured using the Mitosox Red Mitochondrial Superoxide Indicator (Invitrogen). Briefly, CMs were incubated with TMRM/Mitosox and Hoechst 33342 (Beyotime) per manufactures’ instructions, and fluorescence intensities were measured using an Operetta CLS High Content Imaging System (Perkin Elmer).

### Senescence associated-β-galactosidase assay (SA-β-gal)

SA-β-gal activity was assayed as previously reported [28]. Briefly, adipose tissue chunks were collected in PBS, fixed with fixative solution (Solarbio) for 15 min. Then, the adipose tissue chunks were washed 3 times in PBS and placed in SA-β-gal activity solution (Solarbio). After 10–14 h of incubation at 37 °C CO_2_-free incubator, the adipose tissue chunks were rinsed with PBS. Micrographs were captured using an Axio Zoom V16 (ZEISS) microscope and processed in ZEN software (ZEISS).

### miRNAs and RNAs isolation and RT-qPCR

TransZol Up Plus RNA Kit (Trans Gen Biotech) was used for extracting total RNA. RNA reverse transcription was performed by using HiScript II Reverse Transcriptase kit (Vazyme). Real-time quantitative PCR (RT-qPCR) was performed in triplicates using an Applied Biosystems 6 Flex (Thermo Fisher).

The miRNAs were extracted from LEVs using TRIzol reagent (Invitrogen). Briefly, the LEVs were redissolved in 250 μl PBS and mixed with 750 μl TRIzol. Given that U6 mRNA may not be encased within LEVs, caenorhabditis elegans miR-39 (cel-miR-39, 5 nM, RuiboBio) was spiked-in for control. To increase small RNA retrieval, Dr.GenTLE™ Precipitation Carrier (TAKARA) was used per manufacturer's instructions. RT-qPCR was used to determine miRNA levels using miDETECT A TrackTM miRNA RT-qPCR Kit (RuiboBio). The primers for all MicroRNA were obtained from RiboBio Company. The PCR primers are shown in Additional file 7: Table S1.

### RNA library preparation, sequencing, and data analysis

Both mRNA-sequencing and small RNA-sequencing were conducted by Novogene Co., Ltd using the Illumina HiSeq instrument. Briefly, total RNA from AMVMs and adipose derived LEVs were extracted and processed for library construction.

For mRNA-sequencing, RNA integrity was assessed using the RNA Nano 6000 Assay Kit of the Bioanalyzer 2100 system (Agilent). 1 µg total RNA per sample served as input material for the RNA library preparation. A NEBNext^®^ Multiplex RNA Library Prep Set for Illumina^®^ (NEB) was used to create the libraries. Then, sequencing was performed using an Illumina Novaseq platform and 150 bp paired-end reads were captured. For data analysis, image analysis and base calling were performed by the HiSeq Control Software (HCS) + OLB + GAPipeline-1.6 (Illumina) on the HiSeq-instrument. Differential expression analysis was conducted using the DEGseq (2010) R package.

For small RNA-sequencing, a total amount of 3 μg total RNA per sample was used for the small RNA library construction. NEBNext^®^ Small RNA Library Prep Set for Illumina^®^ (NEB) was used to prepare sequencing libraries following the manufacturer’s recommendations. The samples were sequenced using an Illumina Hiseq 2500/2000 platform and 50 bp single-end reads were captured. miRBase20.0 was used as reference, modified software mirdeep2 [32] and srna-tools-cli were used to obtain the potential miRNA and draw the secondary structures. Predicting the target gene of miRNA was performed by miRanda [33] for animals. Differential expression analysis of two conditions/groups was performed using the DESeq R package (3.0.3).

The P-values was adjusted using the Benjamini & Hochberg method. Corrected P-value of 0.05 was set as the threshold for significantly differential expression by default. Differentially expressed genes were further analyzed for pathway enrichment using Ingenuity Pathway Analysis (Qiagen, USA) were right-tailed Fisher’s Exact test.

### Western blot

The total protein was extracted from AMVMs or NMVMs. First, AMVMs or NMVMs were lysed by radioimmunoprecipitation (RIPA) buffer with cocktail protease inhibitor (Beyotime Biotechnology, China) at 4 °C. Then the lysis buffer was clarified by centrifugation at 12,000 rpm for 15 min at 4 °C and the supernatant protein concentration was determined using the bicinchoninic acid method (Beyotime Biotechnology, China). Proteins (20 μg) were size-fractionated by sodium dodecyl sulphate polyacrylamide gel electrophoresis and transferred onto Immobilon polyvinylidene difluoride membranes. The membranes were blocked for 2 h with 5% DifcoTM Skim Milk (BD Biosciences, USA). Then the primary antibodies was added and incubated at 4 °C overnight, and diluted secondary antibody (Cell Signaling Technology, USA) was added and incubated at room temperature for 1 h. The results were visualized and analyzed by Odyssey Infrared Imaging System (LICOR, USA). All primary and secondary antibodies are shown in Additional file 8: Table S2.

### Statistical analysis

Statistical analysis was performed by using GraphPad Prism. All experimental data are presented as the mean ± SEM at least three independent experiments. Statistical analyses were calculated using Student’s t test for two groups, one-way ANOVA followed by Bonferroni post hoc test for multiple groups. P values < 0.05 were considered significant.

## Results

### Surgical removal of senescent epididymal adipose tissue delays the onset of cardiac dysfunction in STZ mice

**Fig. 1:**
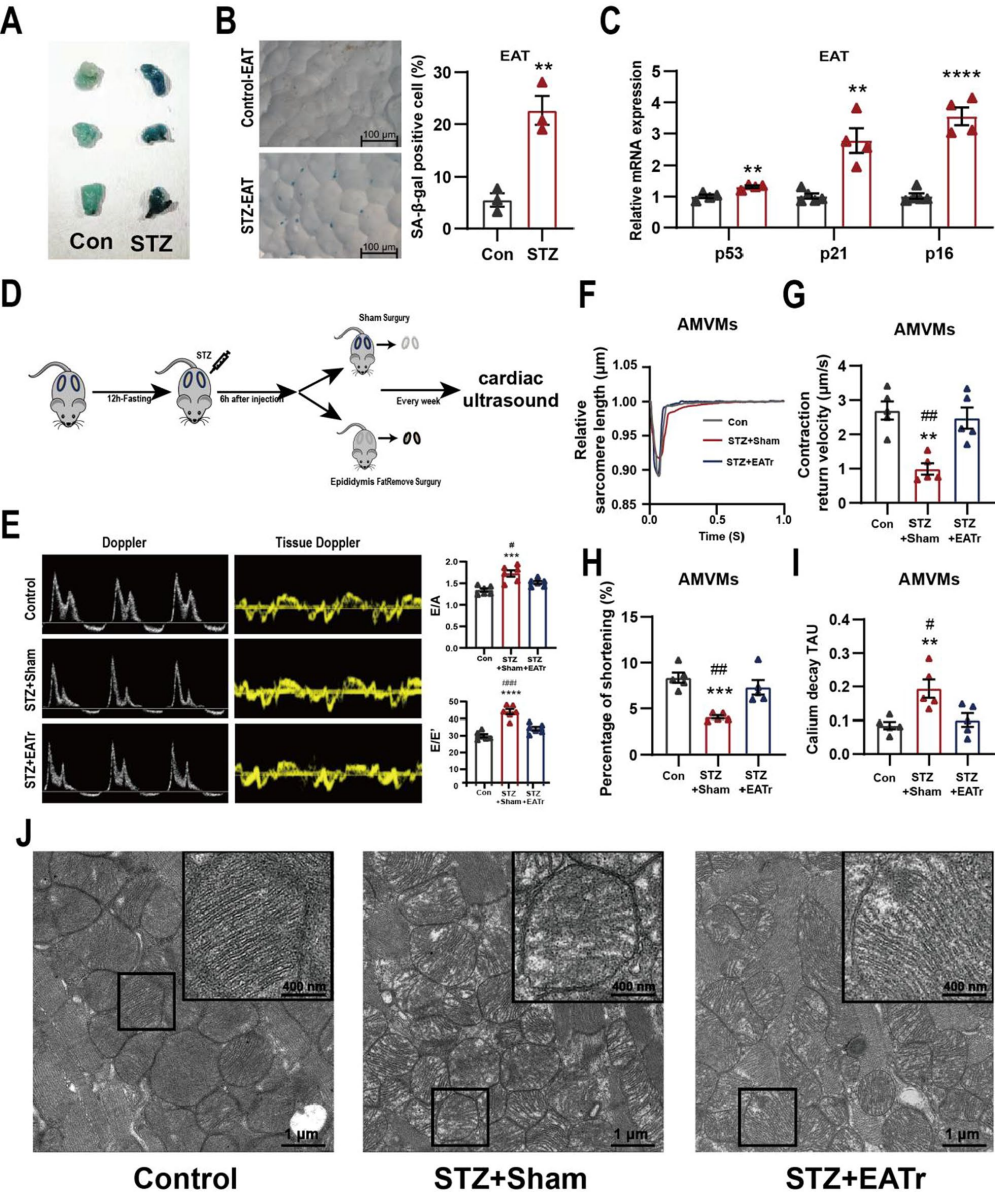
Removal of epididymal adipose tissue alleviates diastolic dysfunction in STZ mice. **A** Representative images of senescence associated beta-galactosidase (SA-β-gal) staining of EAT. **B** Detection of SA-β-gal positive cells from Con- or STZ-EAT (n = 3 per group). **C** Relative gene expression of senescence associated genes in EAT from Con- or STZ-mice (n = 3 per group). **D** Experimental design of EAT removal surgery. **E** Representative of doppler and tissue doppler echocardiography and evaluation of diastolic function (n = 6 per group). **F** Sarcomere length tracing of isolated murine cardiomyocytes using an Ionoptix HTS system. Evaluation of **G** myocardial contraction, **H** fraction shortening and **I** calcium handling in Langendorff-isolated adult mouse ventricular myocytes (AMVMs) (n = 5 per group). **J** Representative micrographs of heart tissue sections examined by transmission electron micrographs. Data are presented as the mean ± SEM; *P < 0.05, **P < 0.01, ***P < 0.001, ****P < 0.0001 compared to Con group; ^#^P < 0.05, ^##^P < 0.01, ^###^P < 0.001 compared to STZ + EATr group

We were surprised to find the presence of adipose tissue senescence in well-established STZ murine model measured by senescence associated β galactosidase (SA-β-gal) staining as well as p53/p21/p16 expression (Fig. 1A–C), similar to what has been observed in adipose tissue from diabetic mice and patients [24, 34]. Surgical removal of senescent adipose tissue in rodents has been shown to prolong lifespan and alleviate myocardial fibrosis [26, 35]. To investigate whether epididymal adipose tissue (EAT) senescence in STZ mice contributes to the development of diabetic cardiomyopathy, we surgically removed epididymal adipose tissue after STZ injection (STZ + EATr) (Fig. 1D). Blood glucose and weight were monitored to ensure the establishment of STZ diabetic model. EATr did not prevent the rise in blood glucose (Additional file 1: Fig. S1A) but mice failed to gain weight like sham surgery counterparts (Additional file 1: Fig. S1B). At 14-weeks, STZ mice with sham surgery (STZ + Sham) developed diastolic dysfunction marked by an increase in E/A ratio and E/E' ratio (Fig. 1E), but showed no difference in ejection fraction (Additional file 1: Fig. S1C) compared to Controls. Surprisingly, STZ + EATr mice failed to develop diastolic dysfunction (Fig. 1E), suggesting EAT senescence may be required for the development of diastolic dysfunction in STZ mice.

Next, we isolated adult mouse ventricular myocytes (AMVMs) using Langendorff perfusion and evaluated contractile function using the Ionoptix HTS system (Additional file 1: Fig. S1D). On single AMVM level, we observed a significant decrease in diastolic velocity as well as fraction shortening (Fig. 1F–H) in STZ + sham AMVMs compared to STZ + EATr and Control AMVMs. Similarly, prolonged calcium reuptake (decay Tau) was observed in STZ + sham AMVMs compared to STZ + EATr and Control AMVMs (Fig. 1I). Using transmission electron microscopy (TEM), we observed disturbed mitochondrial arrangement, mitochondrial swelling, intramitochondrial vacuoles, loss of mitochondrial cristae in STZ + sham compared to control hearts (Fig. 1J). Surprisingly, these mitochondrial remodeling events were absent in STZ + EATr hearts (Fig. 1J). Together, these results demonstrate that EAT senescence is necessary in driving diastolic dysfunction in STZ mice.

### Senescent EAT derived large extracellular vesicles results in cardiomyocyte mitochondrial dysfunction

**Fig. 2:**
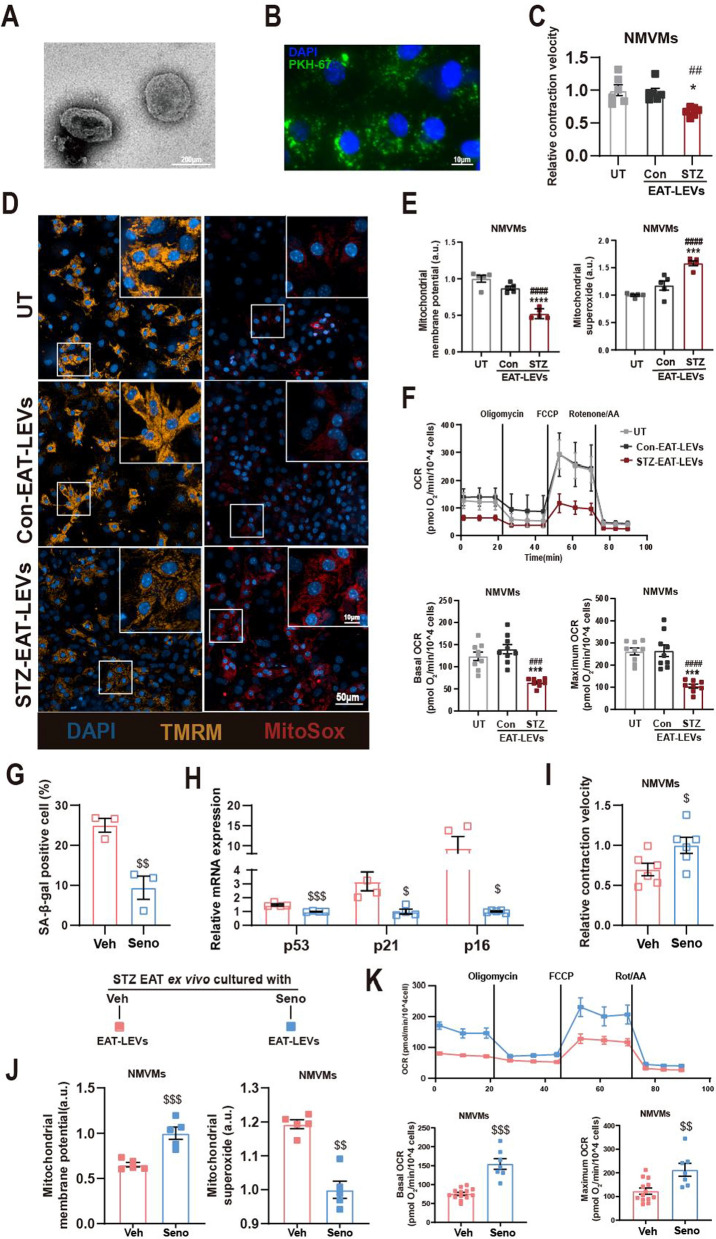
LEVs isolated from senescent EAT in diabetic mice drives contractile and mitochondrial dysfunction in NMVMs. **A** Representative transmission electron micrograph of isolated LEVs. **B** Representative micrograph of EAT derived LEVs (PKH67-labeled, green) co-cultured with NMVMs. **C** Contractile velocity of untreated (UT) or NMVMs treated with Con-EAT LEVs or STZ-EAT LEVs (n = 6 per group). **D** Representative micrograph and **E** quantification of untreated (UT) and NMVMs treated with Con-EAT LEVs or STZ-EAT LEVs stained for mitochondrial membrane potential (TMRM), mitochondrial superoxide (MitoSox), and DAPI (n = 5 per group). **F** Real-time oxygen consumption rates (OCR) were evaluated for untreated (UT) or NMVMs treated with Con-EAT LEVs or STZ-EAT LEVs; basal and maximal respiration rates are shown (n = 8–9 per group). **G** Percentage of SA-β-gal positive cells in STZ-EAT cultured ex vivo in presence of Seno or Veh (n = 3 per group). **H** Relative gene expression of senescence associated genes in STZ-EAT cultured ex vivo in presence of Seno or Veh. (n = 3 per group). **I** Contractile velocity, **J** mitochondrial membrane potential and mitochondrial superoxide relative intensity fluorescence of NMVMs treated with LEVs from STZ-EAT cultured ex vivo in presence of Seno or Veh (n = 5–6 per group). (K) Real-time oxygen consumption rates (OCR) were evaluated for NMVMs treated with LEVs from STZ-EAT cultured ex vivo in presence of Seno or Veh, basal and maximal respiration rates are shown (n = 8–9 per group). Data are presented as the mean ± SEM; *P < 0.05, ***P < 0.001, ****P < 0.0001 compared to UT group; ^##^P < 0.01, ^###^P < 0.001, ^####^P < 0.0001 compared to Con-EAT LEVs group; ^$^P < 0.05, ^$$^P < 0.01, ^$$$^P < 0.001 compared to Veh-STZ-EAT LEVs group

Extracellular vesicles play an important role in inter-organ communication, especially between adipose tissue and distal organs. Characterized by size, large extracellular vesicles (LEVs) are extracellular vesicles with a diameter of 100 nm–1 mm [9]. Using ultracentrifugation, we extracted and characterized LEVs from mouse EAT. TEM images showed that LEVs secreted by EAT had a typical wineglass-like morphology (Fig. 2A) with nanoparticle tracking analysis (NTA) showing that the vast majority of extracted LEVs were above 100 nm in diameter (Additional file 2: Fig. S2A), consistent with the definition of LEVs. Given that AMVMs cannot be cultured for prolong periods, we isolated neonatal mouse ventricular myocytes (NMVMs), as marked by cardiac troponin T (cTnT; Additional file 2: Fig. S2B), as cell model for subsequent studies. To identify whether LEVs from EAT can be absorbed by NMVMs, we treated NMVMs with PKH-67 labeled LEVs. We confirmed that PKH-67 labeled LEVs were taken up by NMVMs within 4 h (Fig. 2B; Additional file 10: Video S1). Next, to understand how STZ-EAT LEVs can modulate myocardial function, we introduced purified LEVs to NMVMs, these NMVMs’ contraction velocity was used as a functional readout using previously established protocol [30, 31]. We found that treatment with STZ-EAT LEVs was able to slow down the contraction velocity of cardiomyocytes compared to untreated group (UT) or LEVs form Con-EAT (Fig. 2C). Proper mitochondrial membrane potential (MMP) is a prerequisite for mitochondrial oxidative phosphorylation and ATP production and was assessed using TMRM dye; excessive mitochondrial superoxide is capable of damaging mitochondria and cardiomyocytes and was stained with MitoSox dye. Using fluorescent probes TMRM and MitoSox, we found that treatment with STZ-EAT LEVs result in decrease of mitochondrial membrane potential and increase of mitochondrial superoxide in NMVMs compared to Con-EAT LEVs (Fig. 2D, E). To further investigate the adverse effects of STZ-EAT LEVs on mitochondrial function in NMVMs, we performed mitochondrial respiration stress test using a Seahorse XFe96 bioanalyzer. We observed that not only the basal mitochondrial respiration but also the maximal respiratory capacity of NMVMs were impaired when NMVMs were co-cultured with STZ-EAT LEVs (Fig. 2F).

Senolytic cocktail, dasatinib plus quercetin, acts through blocking survival pathways while inducing apoptosis in senescent cells [36]. To confirm if LEVs secreted from senescent STZ-EAT is the driver of myocardial dysfunction, we treated isolated STZ-EAT ex vivo with quercetin and dasatinib (Seno-STZ-EAT) or vehicle control (Veh-STZ-EAT). We found that SA-β-gal positive cells in STZ-EAT were significantly reduced after senolytic treatment, and by RT-q-PCR, senescence-related genes (p53/p21/p16) were decreased post senolytic treatment (Fig. 2G, H). Subsequently, we treated NMVMs by LEVs derived from Veh-STZ-EAT or Seno-STZ-EAT, and assayed for contractile and mitochondrial function. Compared to Veh-STZ-EAT LEVs treatment, Seno-STZ-EAT LEVs treated NMVMs exhibited improved contractile function, increased MMP, reduced mitochondrial superoxide as well as improved mitochondrial respiration capacity (Fig. 2I–K) specific to STZ model or related to general adipose senescence, we used doxorubicin (DOX) as a mean to induce adipose senescence in Con-EAT (Veh-Con-EAT versus DOX-Con-EAT). As marked by aging markers, we confirm that DOX treatment is capable of inducing adipose senescence (Additional file 2: Fig. S2C, D). In agreement with our STZ-EAT LEVs results, we found that DOX-Con-EAT LEVs were also capable of decreasing contraction velocity in NMVMs (Additional file 2: Fig. S2E). Mitochondrial function assays also confirmed that DOX-Con-EAT LEVs suppressed myocardial mitochondrial function (Additional file 2: Fig. S2F, G). Together, these results suggests that LEVs derived from senescent adipose tissue can modulate myocardial contraction likely through inhibition of mitochondrial function.

### Identification of miRNA-326-3p and miRNA-339-3p in senescent EAT LEVs

**Fig. 3:**
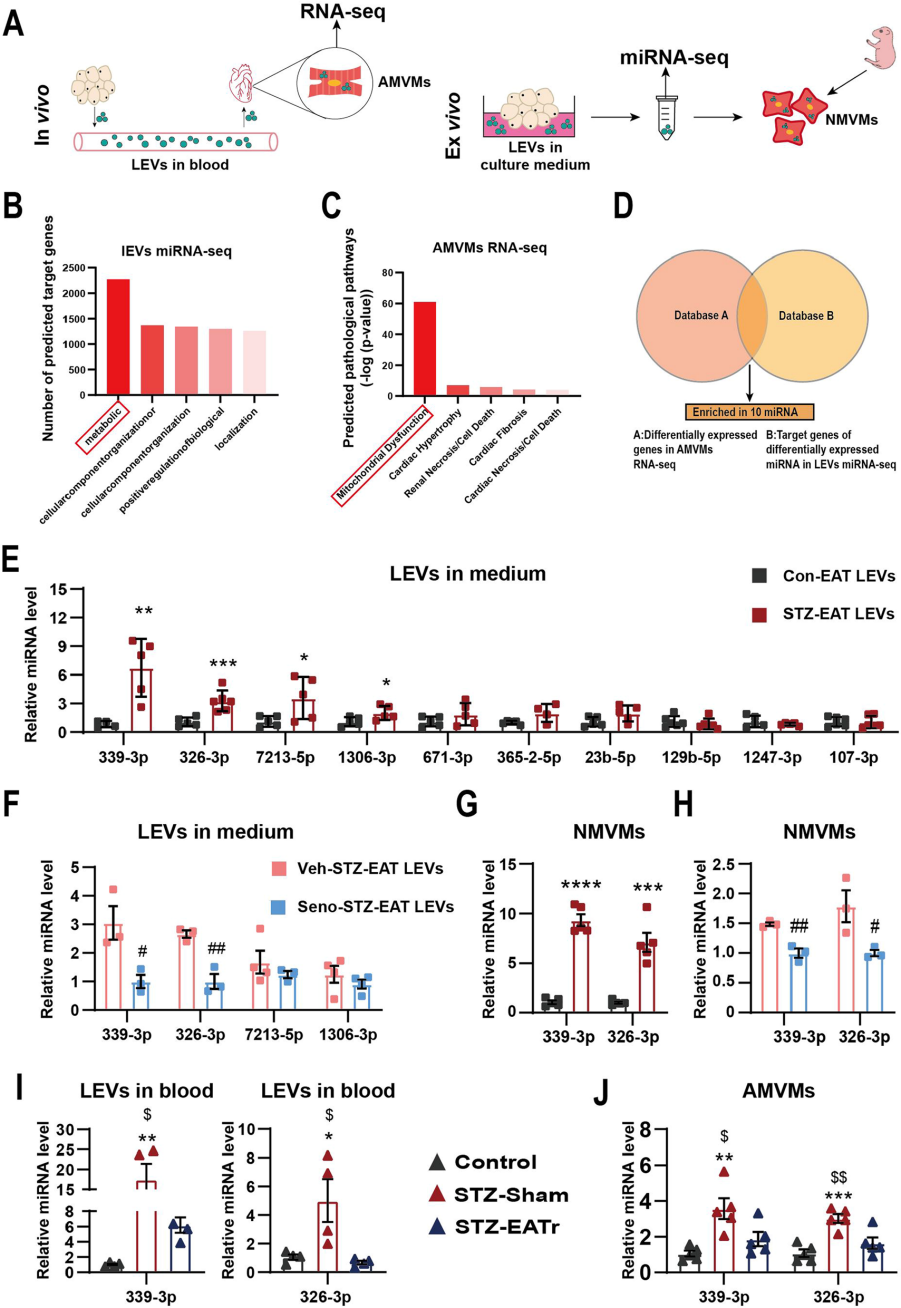
miR-326-3p and miR-339-3p are up-regulated in senescent EAT derived LEVs. **A** Diagram of assays used to identify content encapsulated by senescent EAT derived LEVs in vivo and ex vivo. **B** Gene ontology biological process (BP) enrichment analysis of predicted target genes of differentially expressed LEV-miRNAs. **C** IPA analysis for pathological pathway enrichment of differentially expresses genes in RNA-seq. **D** Representative image of venn analysis between differentially expressed genes in AMVM versus differentially expressed miRNA target genes of LEVs. **E** Expression level of miRNA in EAT culture medium of Con- or STZ- EAT-LEVs (n = 4–6 per group). **F** Expression level of miRNA in Veh or Seno treated STZ-EAT LEVs collected from adipose tissue conditioned medium (n = 3 per group). **G** Expression levels of miRNA-326-3p and miRNA-339-3p in NMVMs treated with Con- or STZ-EAT LEVs (n = 5 per group). **H** Expression level of miRNA in NMVMs treated with LEVs from STZ-EAT cultured ex vivo in presence of Seno or Veh (n = 3 per group). **I** Expression levels of miRNA-339-3p (left) and miRNA-326-3p (right) in murine blood LEVs from control group, STZ + Sham group and STZ + EATr group. (n = 4 per group). **J** Expression levels of miRNA-326-3p and miRNA-339-3p in AMVMs isolated from control group, STZ + Sham group or STZ + EATr group (n = 5 per group). Data are presented as the mean ± SEM; *P < 0.05, **P < 0.01, ***P < 0.001 compared to Con group; ^#^P < 0.05, ^##^P < 0.01 compared to Veh group; ^$^P < 0.05, ^$$^P < 0.01 compared to STZ + EATr group

It has been shown that adipose tissue is the largest source of circulating miRNA [37] responsible for inter-organ communications. Here we postulate that it is the miRNAs encapsulated in LEVs derived from senescent adipose tissue that is responsible for altered myocardial function. To test this postulation, we performed miRNA sequencing on isolated LEVs and RNASeq on isolated AMVMs, respectively (Fig. 3A). First, we identified 36 differentially expressed miRNAs (26 upregulated, 10 downregulated; Additional file 3: Fig. S3A) as well as 1029 differentially expressed genes (687 upregulated, 342 downregulated; Additional file 3: Fig. S3B) in STZ group compared to control group. Next, we examined potential targets of our differentially expressed miRNAs. Using miRBASE and TargetScan database, 36 differentially expressed miRNAs resulted in 2271 predicted targets. GO term enrichment analysis showed that these 2271 predicted target genes were enriched for metabolism-related functions (Fig. 3B); furthermore, IPA GO term analysis of the 1029 differentially expressed genes from RNASeq were enriched for metabolic diseases—the most prominent being mitochondrial oxidative phosphorylation (Figs. 3C and Additional file 3: Fig. S3C). Next, we performed a venn diagram comparison between our differentially expressed miRNAs in LEVs to differentially expressed genes in AMVMs (Fig. 3D). Total of 160 genes (4 upregulated and 154 downregulated) served as input back into miRBASE and TargetScan database to generate a list of potential miRNAs. By comparing the predicted miRNAs with our differentially expressed miRNAs, we identified total of 3 downregulated miRNAs (targeting 4 upregulated genes) and 10 upregulated miRNAs (targeting 154 downregulated genes. Next, we focused our validation efforts on up-regulated miRNAs/downregulated targets. We carried out our candidate validation with the following criteria: the upregulated miRNA candidate must be expressed and secreted in LEVs by senescent adipose tissue, present in circulation, absorbed by AMVMs in vivo and must be present in LEVs secreted by ex vivo cultured adipose tissue and that can also be absorbed by NMVMs (Fig. 3A). Using RT-qPCR, we confirmed that miRNA-339-3p, miRNA-326-3p, miRNA-7213-5p and miRNA-1306-5p are significantly expressed in STZ-LEVs secreted by STZ EAT compared to LEVs collected from Control EAT (Fig. 3E).

To investigate whether these up-regulated miRNAs were secreted by senescent cells in EAT, we measured our four miRNA candidates in STZ-EAT ex vivo treated with senolytic and Con-EAT ex vivo treated with DOX. Out of the four candidates, only miRNA-339-3p and miRNA-326-3p were significantly decreased in Seno-STZ-EAT LEVs compared to Veh-STZ-EAT LEVs (Fig. 3F); meanwhile miRNA-339-3p and miRNA-326-3p were significantly increased in DOX-Con-EAT LEVs compared to Veh-Con-EAT LEVs (Additional file 3: Fig. S3D). Next, we wanted to test if miRNA-339-3p and miRNA-326-3p can be detected in cardiomyocytes after LEVs have been absorbed by NMVMs. Indeed, after treating with STZ-EAT LEVs, we were able to detect the increase in miRNA-326-3p and miRNA-339-3p in NMVMs compared to Con-EAT LEVs treatment (Fig. 3G); this increase was lost if the STZ-EATs were treated with senolytic prior to LEV isolation and co-culture with NMVMs (Fig. 3H). Moreover, we were able to recapitulate this observation in Dox-Con-EAT LEVs system (Additional file 3: Fig. S3E). Next, we went back in vivo and verified that indeed miRNA-326-3p and miRNA-339-3p were significantly enriched in LEVs isolated from STZ + sham blood compared to Control or STZ + EATr blood samples (Fig. 3I); similar trend was observed in AMVMs isolated from these mice (Fig. 3J). Together, these data show that miRNA-326-3p and miRNA-339-3p are the key adipose derived senescence relevant miRNAs that is responsible for diastolic dysfunction in STZ hearts.

### miRNA-326-3p lead to contractile and mitochondrial dysfunction in cardiomyocytes by inhibiting Rictor

**Fig. 4:**
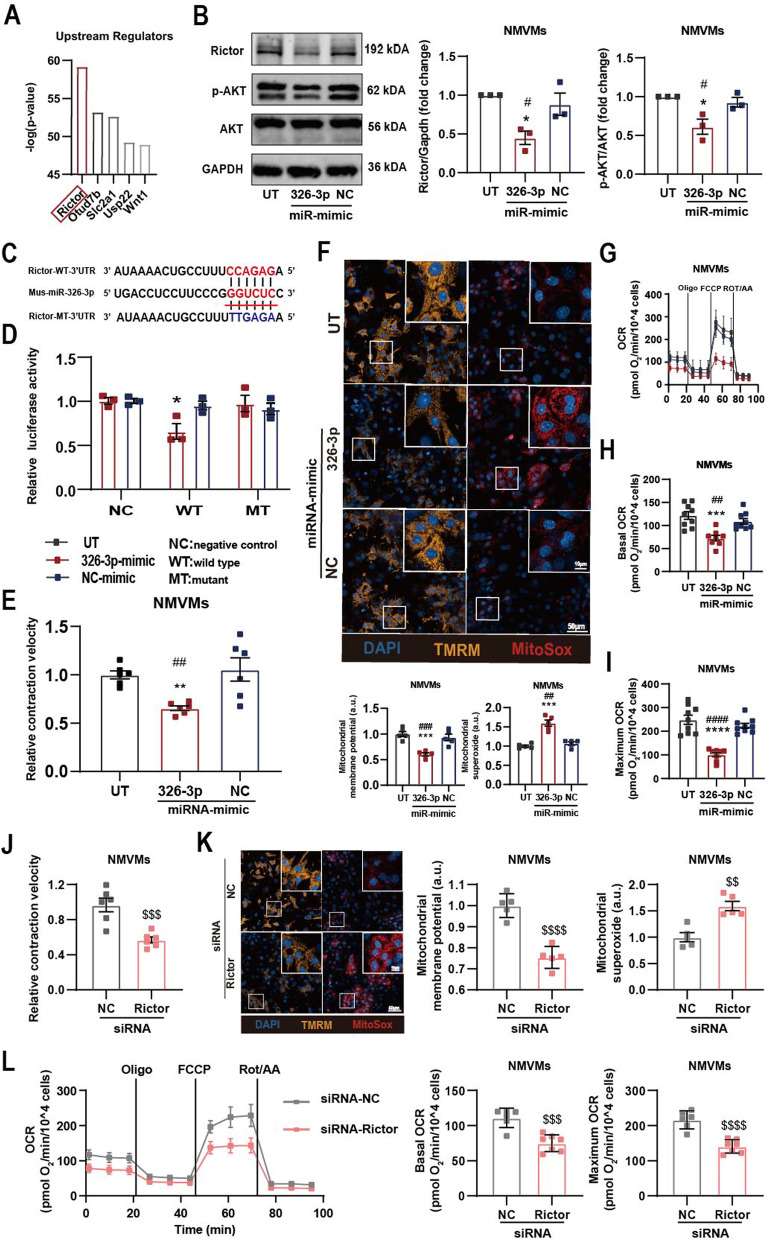
miRNA-326-3p decreases NMVMs’ contractile and mitochondrial function by inhibiting Rictor. **A** Predicted upstream regulators of differentially expressed genes in AMVMs isolated from STZ mice by IPA analysis. **B** Western blotting analysis of Rictor, p-AKT, AKT and Gapdh in UT or NMVMs transfected with 326-3p- or negative control- (NC-) miR-mimic. (n = 3 per group). **C** Schematic diagram showing binding of miR-326-3p to 3′-UTR of Rictor. Complementary sequences are highlighted in red, mutated sequence in blue. **D** Luciferase reporter assays performed with firefly luciferase under control of the Rictor 3′ UTR (wild type (WT) or mutant (MT)), control Renilla luciferase and the miR-326-3p (n = 3 per group). **E** Contractile velocity of UT or NMVMs transfected with 326-3p- or NC-miR-mimic (n = 6 per group). **F** Representative micrographs of UT or NMVMs, transfected with 326-3p- or NC-miR-mimic, stained for mitochondrial membrane potential (TMRM), mitochondrial superoxide (MitoSox), and DAPI, mitochondrial membrane potential and mitochondrial superoxide relative intensity of fluorescence are shown (n = 5 per group). **G** Real-time oxygen consumption rates (OCR) were evaluated for UT or NMVMs transfected with 326-3p- or NC-miR-mimic, Basal (**H**) and maximal (**I**) respiration rates are shown (n = 8–9 per group). **J** Contractile velocity of NMVMs transfected with Rictor- or NC-siRNA (n = 6 per group). **K** Representative micrograph and fluorescence intensity quantification of NMVMs transfected with Rictor- or NC-siRNA and stained for mitochondrial membrane potential (TMRM) or mitochondrial superoxide (MitoSox) and DAPI (n = 5 per group). **L** Real-time oxygen consumption rates (OCR) were evaluated for NMVMs transfected with Rictor- or NC-siRNA; basal and maximal respiration rates are shown (n = 6 per group). Data are presented as the mean ± SEM; *P < 0.05, **P < 0.01, ***P < 0.001, ****P < 0.0001 compared to UT group; ^#^P < 0.05, ^##^P < 0.01, ^###^P < 0.001, ^####^P < 0.0001 compared to NC-miR-mimic group; ^$^P < 0.05, ^$$^P < 0.01, ^$$$^P < 0.001, ^$$$$^P < 0.0001 compared to INC-siRNA group

As mentioned above, mitochondrial dysfunction was the most significant pathological alteration in STZ diabetic AMVMs based on our bioinformatic analyses. We asked whether there is a key pathway targeted by miRNA-326-3p and miRNA-339-3p that is upstream of other differentially expressed genes. Using IPA enrichment analysis, we found that Rictor (targetd by miRNA-326-3p), an essential protein of mammalian target of rapamycin complex 2 (mTORC2), was likely the upstream regulator of STZ myocardial pathology (Fig. 4A). To verify whether miRNA-326-3p inhibits Rictor expression, we transfected a miRNA-326-3p mimic or negative control (NC) into NMVMs and verified transfection efficiency by RT-qPCR (Additional file 4: Fig. S4A). We observed that both mRNA and protein level of Rictor was reduced in NMVMs overexpressing miRNA-326-3p (Additional file 4: Fig. S4B; Fig. 4B). For downstream validation, we used reduced AKT phosphorylation as a readout of decreased mTORC2 activation (Fig. 4B). To demonstrate direct regulation of Rictor by miRNA-326-3p, we generated firefly luciferase reporters under control of the wild type (WT) Rictor 3′UTR or mutated (MT) sequence. Using this system, we found that mouse wide type Rictor (WT-Rictor) 3′UTR luciferase activity was significantly inhibited upon overexpression of miRNA-326-3p mimic; the inhibitory effect was abolished when the binding site was mutated (MT-Rictor; Fig. 4C, D).

Next, we asked if the identified miRNA-326-3p-Rictor axis was responsible for the observed decline in myocardial and mitochondrial function. At the cellular level, we found that transfecting miRNA-326-3p mimic into NMVMs resulted in a decrease in contraction function (Fig. 4E), a decrease in mitochondrial membrane potential, an increase in mitochondrial superoxide and a decrease in mitochondrial respiration (Fig. 4F–I). To show it is indeed Rictor that is responsible for the functional decline, we transfected siRNA-Rictor into NMVMs with good knockdown efficiency at the protein level (Additional file 4: Fig. S4C). We found that knockdown of Rictor in NMVMs resulted a decrease in contraction function (Fig. 4J), a decrease in mitochondrial membrane potential and an increase in mitochondrial superoxide (Fig. 4K) and a decline in mitochondrial respiratory function (Fig. 4L). These data are in keep with our miRNA-326-3p overexpression observations.

**Fig. 5:**
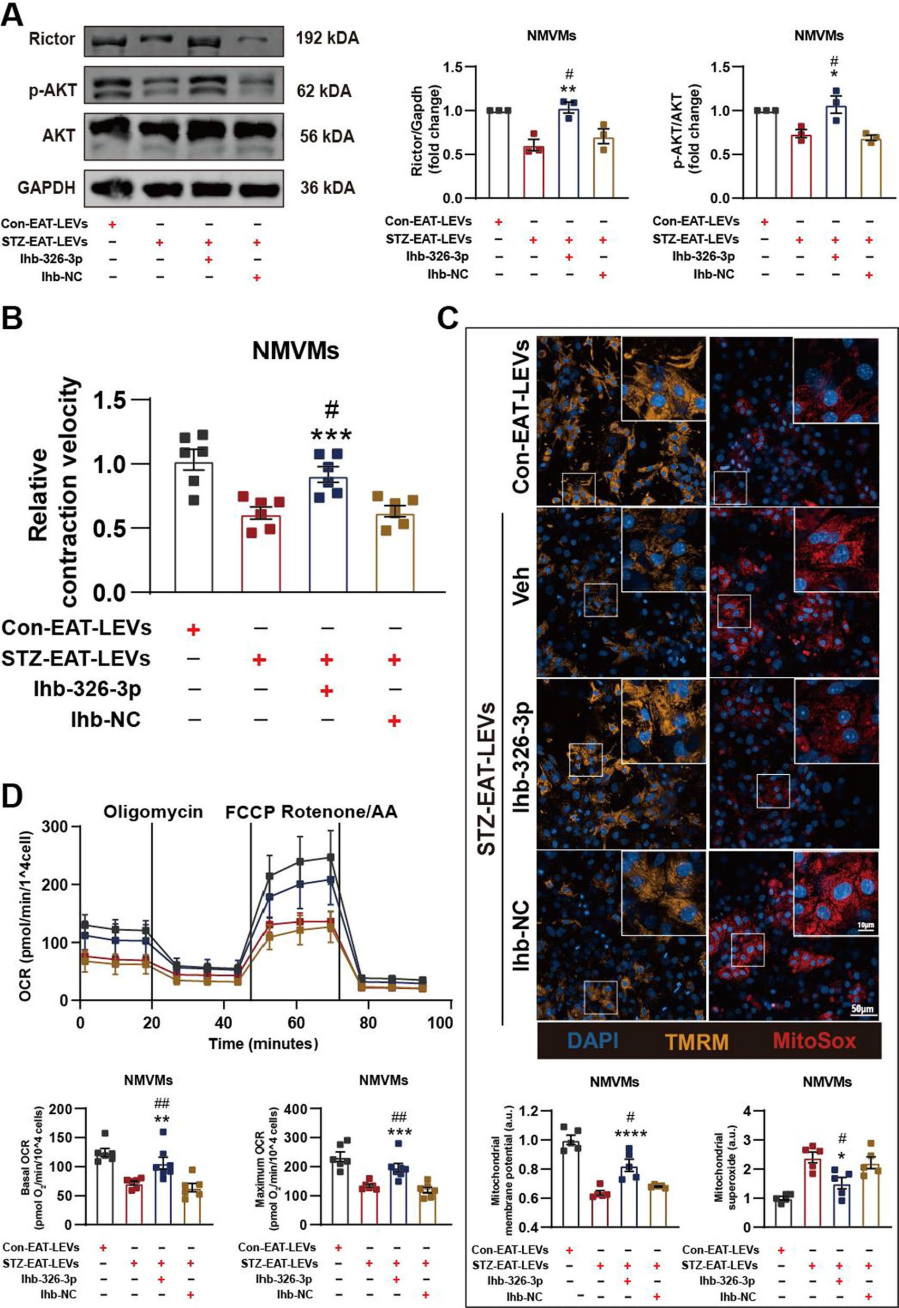
Senescent EAT derived LEVs repress NMVMs Rictor expression and cause NMVMs dysfunction via miRNA-326-3p. **A** Western blotting analysis of Rictor, p-AKT, AKT and Gapdh in Con- or STZ-EAT LEVs co-cultured NMVMs transfected with miR decoy (Inh-326-3p) or negative decoy control (Inh-NC) (n = 3 per group). Con- or STZ-EAT LEVs co-cultured NMVMs were transfected with Inh-326-3p or Inh-NC and **B** contractile function (n = 6 per group), **C** mitochondrial membrane potential (TMRM) and mitochondrial superoxide (MitoSox) (n = 5 per group), and **D** mitochondrial respiration (n = 6 per group) were assayed. Data are presented as the mean ± SEM; *P < 0.05, **P < 0.01, ***P < 0.001, ****P < 0.0001 compared to STZ-EAT LEVs group; ^#^P < 0.05, ^##^P < 0.01 compared to Inh-NC group

Lastly, we transfected NMVMs with miRNA-326-3p decoy (Inh-326-3p) or negative control (NC) prior to various LEV treatments. As predicted, presence of miRNA-326-3p decoy prevented downregulation of Rictor protein in NMVMs were treated with STZ-EAT or DOX-Con-EAT LEVs (Fig. 5A; Additional file 5: Fig. S5A), prevented decrease in contraction function Fig. 5B; Additional file 5: Fig. S5B), mitochondrial dysfunction (Fig. 5C, D; Additional file 5: Fig. S5C, D). Together, these data demonstrate that miRNA-326-3p-Rictor pathway is responsible for mitochondrial and contraction dysfunction caused by STZ-EAT LEVs in cardiomyocytes.

### Senolytic treatment alleviates diastolic dysfunction in STZ mice

**Fig. 6:**
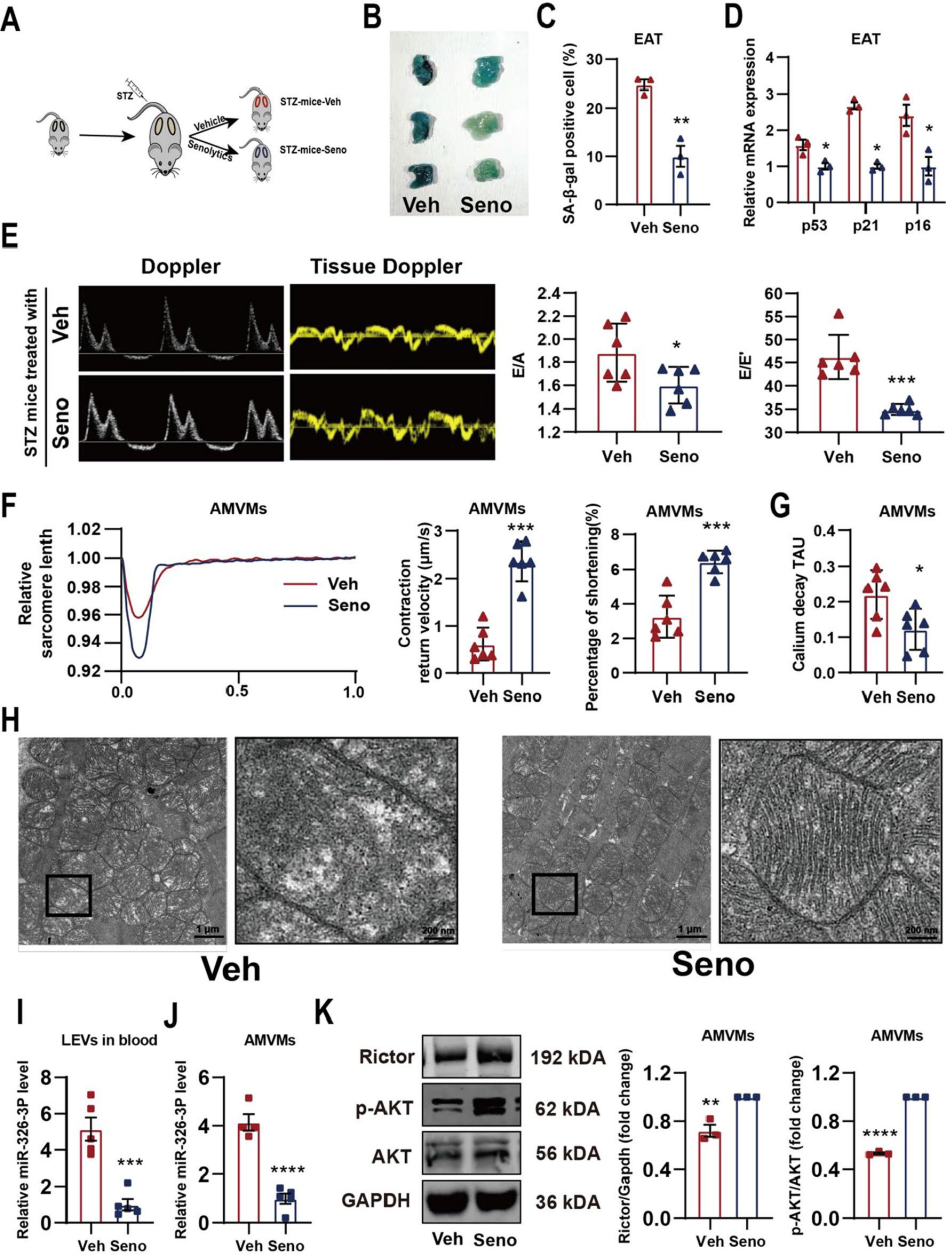
Senolytic treatment alleviates cardiac function in STZ mice. **A** Experiment design for senolytic treatment in vivo. **B** Representative images and **C** quantification of (SA-β-gal) staining of EAT isolated from Veh or Seno treated STZ-mice (n = 3 per group). **D** Relative gene expression of senescence associated genes in Veh or Seno treated STZ-EAT (n = 3 per group). **E** Representative of doppler and tissue doppler echocardiography and evaluation of diastolic function (n = 6 per group). **F** Contractile function and **G** calcium handling of AMVMs isolated from Veh or Seno treated STZ-mice were evaluated (n = 5 per group). **H** Representative transmission electron micrographs of Veh or Seno treated STZ-mice hearts. Relative miRNA-326-3p expression in **I** blood and **J** AMVMs from Veh or Seno treated STZ-mice (n = 4–5 per group). **K** Protein levels of Rictor, p-AKT, AKT and Gapdh in AMVMs isolated from Veh or Seno treated STZ-mice evaluated by immunoblotting (n = 3). Data are presented as the mean ± SEM; *P < 0.05, **P < 0.01, ***P < 0.001, ****P < 0.0001 compared to Veh group

**Fig. 7:**
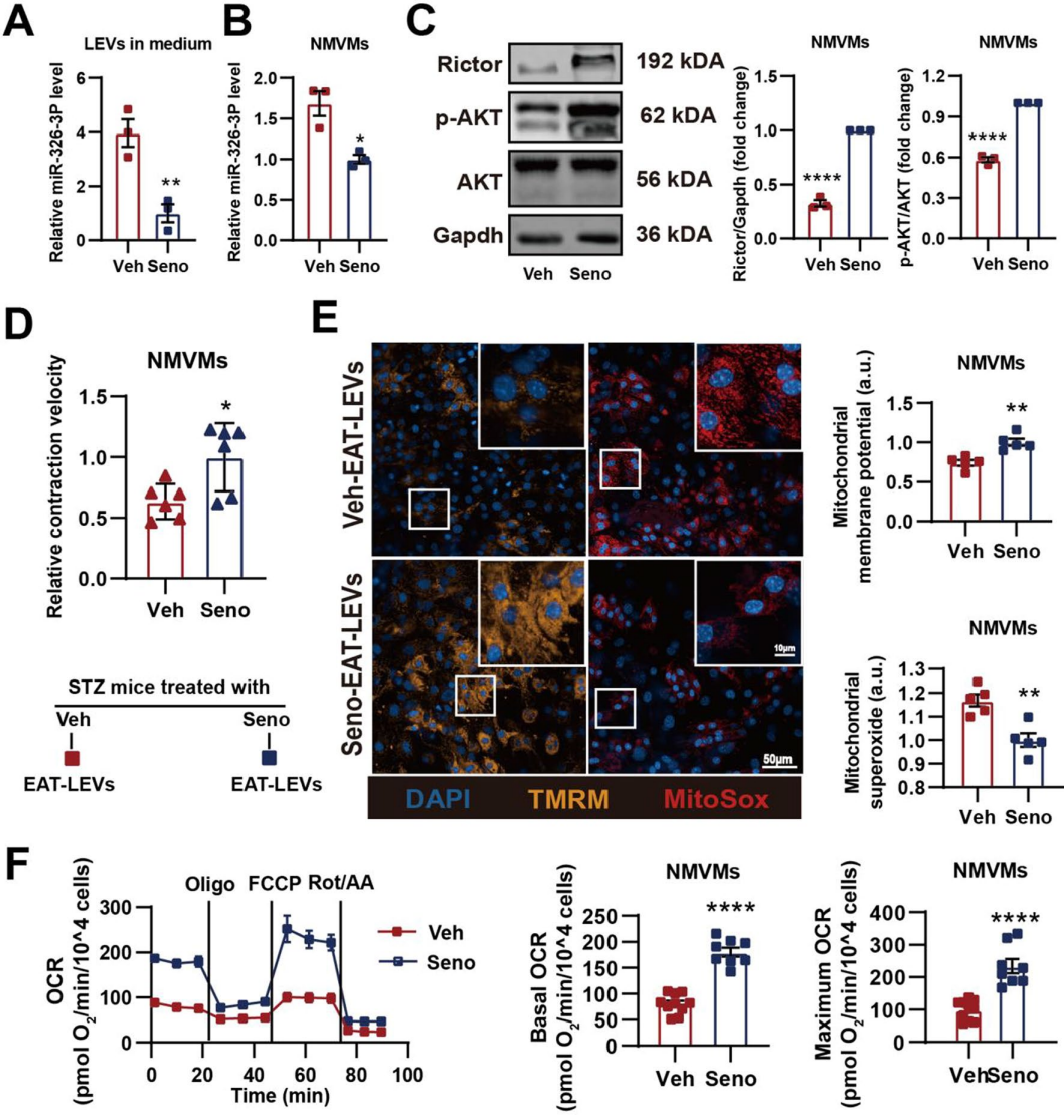
Senolytic reduces miRNA-326-3p containing LEVs secretion from senescent EAT and prevents onset of mitochondrial dysfunction. **A** Relative expression level of miRNA-326-3p in EAT-LEVs collected from vehicle or senolytic treated STZ-mice (n = 3 per group). **B** Expression level of miRNA-326-3p in NMVMs treated with STZ-seno- or STZ-veh-EAT LEVs (n = 3 per group). **C** Western blotting analysis of Rictor, p-AKT, AKT and Gapdh in STZ-seno- or STZ-veh-EAT LEVs treated NMVMs (n = 3 per group). NMVMs treated with STZ-seno- or STZ-veh-EAT LEVs and **D** contractile function (n = 5 per group), **E** mitochondrial membrane potential (TMRM) and mitochondrial superoxide (MitoSox) (n = 5 per group), **F** mitochondrial respiration (n = 8–9 per group) were evaluated. Data are presented as the mean ± SEM; *P < 0.05, **P < 0.01, ***P < 0.001, ****P < 0.0001 compared to Veh group

Using the previously established senolytic regime [38], we subjected STZ mice with dasatinib plus quercetin or vehicle via oral gavage (Seno-STZ-mice versus Veh-STZ-mice) to test if senolytic can reduce EAT senescence and alleviate diabetic cardiomyopathy through inhibition of miRNA-326-3p-Rictor pathway (Fig. 6A). We found that senolytic treatment in vivo indeed lowered adipose senescence burden (Fig. 6B–D) and alleviated diastolic dysfunction (Fig. 6E) but had no effect in systolic function (Additional file 6: Fig. S6A). At the cellular level, we observed that myocardial contractility (Fig. 6F) and calcium handling (Fig. 6G) significantly improved in AMVMs isolated from senolytic treated STZ mice. At the mitochondria level, senolytic treatment prevented the development of mitochondrial ultrastructure changes in STZ hearts compared to vehicle treated STZ hearts (Fig. 6H). Next, we wanted to examine if senolytic treatment blocked the expression and secretion of miRNA-326-3p in LEVs. Compared to Veh-STZ-mice, miRNA-326-3p was significantly decreased in LEVs isolated from circulation and isolated AMVMs from Seno-STZ-mice (Fig. 6I, J). Align with reduced miRNA-326-3p, Rictor protein levels as well as phosphorylated AKT proteins were preserved in AMVMs from Seno-STZ hearts compared to Veh-STZ hearts (Fig. 6K). In contrast where we treated STZ-EAT with senolytic ex vivo, here we administered senolytic in vivo and then isolated LEVs for NMVM co-culture experiments. In accordance, we confirmed that senolytic treatment in vivo inhibited miRNA-326-3p expression in LEVs (Fig. 7A) and in NMVMs (Fig. 7B), In line with down-regulated miRNA-326-3p, Rictor expression and AKT phosphorylation level were preserved in NMVMs when treated with LEVs from Seno-STZ-mice EAT compared to Veh-STZ-mice EAT (Fig. 7C), restored myocardial contraction (Fig. 7D) and mitochondrial function in NMVMs (Fig. 7E, F). Together, these data demonstrate that in vivo senolytic treatment is capable of blocking the miRNA-326-3p-Rictor pathway activation and restore cardiac function in STZ mice.

## Discussion

**Fig. 8:**
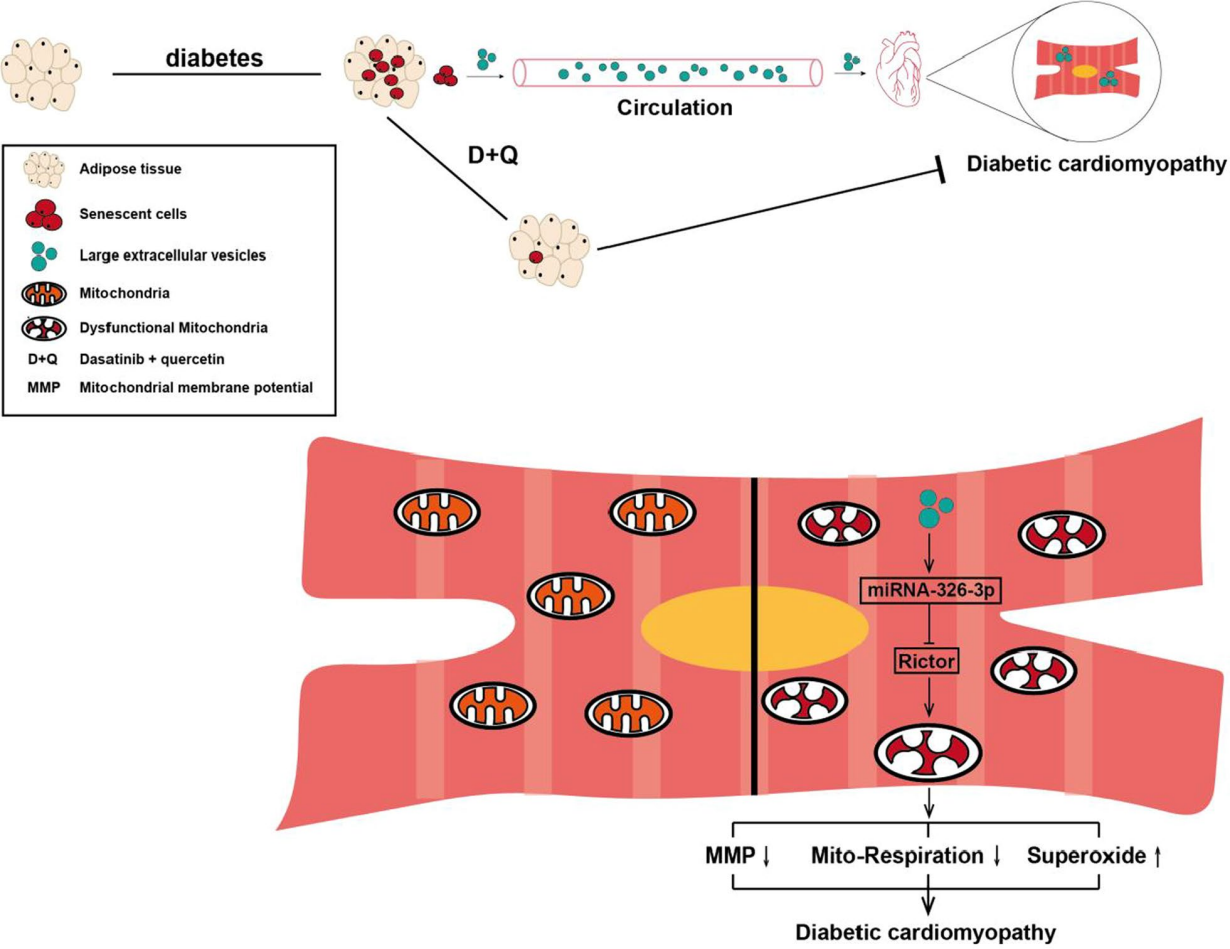
Scheme of the mode of senescent fat exacerbates myocardial metabolism in diabetic mice

There has been long-lasting agreement on the negative impact of obesity on health and the increase in risk of diabetes. However what pathological remodeling occurs in diabetes adipose and the mechanisms by which adipose tissue promotes diabetic cardiomyopathy have not been fully elucidated. Here we demonstrate that adipose senescence in STZ diabetic mice is responsible for decreased cardiac functions via inter-organ miRNA-326-3p-Rictor pathway. Removal of senescent cells in adipose tissue, albeit surgically or pharmacologically, is capable of alleviating diabetic cardiomyopathy. Mechanistically, our work demonstrates that senescent cells in adipose tissue secrete miRNA-326-3p containing LEVs and orchestrates a fat-heart metabolic remodeling through downregulation of myocardial Rictor protein (Fig. 8).

Dysfunctional adipose secrete EVs promotes insulin resistance, deteriorates liver function as well as tumor cell metabolism [14, 39, 40]. Recent studies show that SEVs secreted by dysfunction adipose tissue increase myocardial ischemia–reperfusion injury [41]. Here we show that adipose senescence induces myocardial metabolic remodeling and results in diastolic dysfunction. Surgical removal of EAT in STZ mice prevented the onset of diastolic dysfunction in STZ mice; contractile function, calcium handing, and mitochondrial ultrastructures were preserved in STZ + EATr-AMVMs. Mitochondrial involvement in the development of diabetic cardiomyopathy continues to be an active field [42, 43]. Aside from producing ATP, mitochondria can also be a major source of ROS in diabetic cardiomyocytes [44]. The impermeability of the inner mitochondrial membrane is essential for maintaining electrochemical potential difference that drives ATP synthesis; high mitochondrial ROS can increase the permeability of the inner mitochondrial membrane leading to a decrease in membrane potential that results in a decrease in ATP production [45]. In our ex vivo experiment*,* LEVs derived from STZ-EAT increased mitochondrial ROS and decreased MMP as well as mitochondrial respiration. Whether STZ-EAT LEVs drives diastolic dysfunction through low ATP availability, perhaps similar to familial hypertrophic cardiomyopathy [46], or involves other complications with glucose, which can be attenuated by SGLT2 inhibitors [47], requires further investigation.

It has been shown that hyperglycemia in STZ-diabetic mice exhibit an increase in advanced glycation end products (AGE) increases oxidative stress and can trigger endothelial and vascular smooth muscle cells senescence [48, 49]. We speculate that oxidative stress-induced DNA damage in STZ animals is responsible for driving adipose senescence; this is supported by our results where Dox treated adipose tissue can also secrete miRNA-326-3p containing LEVs to drive metabolic dysfunction in cardiomyocytes. Previously, it has been shown that adipose tissue is one of the earliest organs to show signs of senescence due to natural aging [50] or premature senescence [25]; senescent adipose results in metabolic disorders [34], adipose inflammation and increase in circulatory SASP [51]. Here, we show that LEVs secreted by senescent adipose drives myocardial metabolic remolding via induction of mitochondrial ROS as well as reduction of ATP production. Interestingly, transverse aortic constriction (TAC) can result in adipose p53-dependent insulin resistance [52]. Given the accumulating evidence, we propose that targeting the pathogenic adipose-heart signaling axis may be desirable for treating metabolic diseases. Although we have demonstrated that administration of senolytic can prevent the onset of diastolic dysfunction in STZ animals, one should caution the dosage and duration given that systemic removal of senescent cells may trigger fibrosis as part of natural tissue damage response. Localized administration of senolytic may be desirable [25] prevention of adipose senescence and may prevent the deterioration of other aging organs [23].

Lastly, we identified that miRNA-326-3p and miRNA-339-3p expression is up-regulated in senescent adipose tissue, packaged, and secreted into circulation, and is taken up by cardiomyocytes. miRNA containing EVs secreted by senescent cells play as SASP mediators in senescent environment [53, 54]. Given that adipose secrete more than 60% of miRNA in circulation, we propose that senescent adipose may regulate remote organs metabolism partially via senescence relevant miRNA. Although our analyses suggest miRNA-339-3p may be involved in the regulation of myocardial fibrosis (data not shown), we choose and characterized the miRNA-326-3p-Rictor signaling axis based on our bioinformatic analyses suggesting metabolic dysregulation. We demonstrate that miRNA-326-3p drives mitochondrial dysfunction in NMVMs through inhibiting the translation of Rictor. Although mTORC2 is associated with cell metabolism [55, 56] yet its role in cardiomyocytes remains to be explored. Given that Rictor is at the heart of the mTORC2 complex, we speculate based on previous work that loss of Rictor in STZ murine cardiomyocytes likely results in attenuation of metabolite influx into mitochondria [57], blocked connexin 43 localization to mitochondria [58], and mitochondrial dysfunction [59]. There are two distinct mTOR complexes, mTORC1 and mTORC2, that function very differently. mTORC1 and its inhibitor rapamycin have been extensively shown to be beneficial in regulating metabolism and extending lifespan [60]. Given that Rictor is cardioprotective and its mRNA is targeted by miRNA-326-3p, we speculate restoration of Rictor may be capable of preventing metabolic remolding during DCM or cardiac aging.

One limitation of our study is that we can only demonstrate the dark side of premature adipose tissue in STZ type 1 diabetic mice which cannot elucidate the harmful effects of adipose tissue during nature aging. Geriatric animal models are needed to further explore this issue in future studies. Another limitation of our study is that we have not clarify the subtype of senescent cells in diabetic adipose tissue that derive senescence relevant miRNA. Further explore of this issue may provide a more precise therapeutic target preventing harmful fat-heart signaling in diabetes.

## Conclusion

Here we identified that senescent adipose tissue can regulated myocardial metabolism through the secretion of miRNA-326-3p. Intervention, either through surgical removal or administration of senolytic prevents the presence of miRNA-326-3p containing LEVs in circulation, loss of myocardial Rictor, and prevents the onset of diabetes induced diastolic dysfunction. Our results warrant for further validation if miRNA-326-3p as a potential target for other heart failure models and demonstrate the presence of fat-heart crosstalk in diabetic cardiomyopathy.

## Supplementary Information

**Additional file 1: Figure S1.** Blood glucose and weight monitoring of experimental mice. (A) Blood glucose tracking of mice after STZ injection. (B) Weight monitoring over time of mice after STZ injection. (C) Representative echocardiography M-model of long axis view used to evaluate ejection fraction (n = 6 per group). (D) Representative image of Langendorff-purified adult mouse ventricular myocytes (AMVMs).

**Additional file 2: Figure S2.** LEVs secreted by senescent EAT impair mitochondrial functions in NMVMs. (A) Distribution analysis of particle size for LEVs using nanosight tracking analysis. (B) Representative micrograph in which NMVMs were stained with cardiac troponin T and DAPI. (C) Detection of SA-β-gal activity in Con-EAT cultured ex vivo in presence of DOX or Veh (n = 3 per group). (D) Relative gene expression of age-associated genes in Con-EAT cultured ex vivo in presence of DOX or Veh (n = 3 per group). (E) Contractile velocity of NMVMs treated with LEVs from Con-EAT cultured ex vivo in presence of DOX or Veh (n = 6 per group). (F) Representative micrograph of NMVMs treated with LEVs from Con-EAT cultured ex vivo in presence of DOX or Veh and stained for mitochondrial membrane potential (TMRM) or mitochondrial superoxide (MitoSox) and DAPI, mitochondrial membrane potential and mitochondrial superoxide relative intensity of fluorescence are shown (n = 5 per group). (G) Real-time oxygen consumption rates (OCR) were evaluated for NMVMs treated with LEVs from Con-EAT cultured ex vivo in presence of DOX or Veh, basal and maximal respiration rates are shown (n = 8 per group). Data are presented as the mean ± SEM; *P < 0.05, **P < 0.01, ****P < 0.0001 compared to Veh group.

**Additional file 3: Figure S3.** miRNA-seq of LEVs and mRNA-seq of AMVMs and RT-qPCR verification for DOX-EAT LEVs. (A) LEVs from Con-EAT and STZ-EAT were purified and subjected to non-coding RNA-Seq. Heat map of differentially expressed LEV-miRNAs derived form Con- or STZ-EAT are shown. (B) Volcano plot of differentially expressed genes from isolated AMVMs RNA-seq. (C) Diagram of mitochondria-related signaling pathways, green = up-regulated different genes, red = down-regulated genes. (D) Expression level of miRNA in Veh- or DOX-Con-EAT LEVs collected from adipose tissue conditioned medium (n = 5 per group). (E) Expression level of miRNA-339-3p and miRNA-326-3p in NMVMs treated with LEVs from Con-EAT cultured ex vivo in presence of DOX or Veh (n = 5 per groups). Data are presented as the mean ± SEM; ###P < 0.001, ####P < 0.0001 compared to Veh group.

**Additional file 4: Figure S4.** Characterization of miRNA-326-3p mimic transfection efficiency in NMVMs. (A) Relative miRNA-326-3p expression level in UT or NMVMs transfected with 326-3p- or NC-miR-mimic (n = 3 per group). (B) Relative Rictor mRNA expression level in UT or NMVMs transfected with 326-3p- or NC-miR-mimic (n = 3–6 per group). (C) Protein levels of Rictor, p-AKT, AKT and Gapdh in NMVMs transfected with Rictor- or NC-siRNA. (n = 3 per group). Data are presented as the mean ± SEM; *P < 0.05, **P < 0.01 compared to UT group; #P < 0.05 compared to NC-miR-mimic group; $$P < 0.01, $$$$P < 0.0001 compared to NC-siRNA group.

**Additional file 5: Figure S5.** DOX-induced senescent EAT derived LEVs repress NMVMs Rictor expression via miRNA-326-3p. (A) Protein levels of Rictor, p-AKT, AKT and Gapdh in DOX-EAT LEVs co-cultured NMVMs transfected with miR decoy (Inh-326-3p) were evaluated by immunoblotting. (n = 3 per group). DOX-EAT LEVs co-cultured NMVMs were transfected with Inh-326-3p or untreated and (B) contractile velocity (n = 6 per group), (C) mitochondrial membrane potential (TMRM) and mitochondrial superoxide (MitoSox) (n = 5 per group), and (D) mitochondrial respiration (n = 6 per group) were assayed. Data are presented as the mean ± SEM; $P < 0.05, $$P < 0.01, $$$P < 0.001, $$$$P < 0.0001 compared to DOX-EAT LEVs group.

**Additional file 6: Figure S6.** Preservation of cardiac systolic function in STZ mice treated with senolytic. (A) Representative M-model long axis echocardiography and ejection fraction (EF%) of Seno or Veh treated STZ mice (n = 6 per group).

**Additional file 7: Table S1.** Primers used in experiment

**Additional file 8: Table S2.** Antibodies used in experiment.

**Additional file 9: Table S3.** Reagent or resource used in experiment.

**Additional file 10: Video S1.** PKH-67 labeled LEVs were taken up by NMVMs.

## Data Availability

The datasets used and analysed during the current study are available from the corresponding author on reasonable request.

## Abbreviations

DCM: Diabetic cardiomyopathy
STZ: Streptozotocin
AMVMs: Adult mouse ventricular myocytes
NMVMs: Neonatal mouse ventricular myocytes
SA-β-gal: Senescent associated β galactosidase
LEVs: Large extracellular vesicles
EVs: Extracellular vehicles
SASP: Senescence-associated secretory phenotype
EATr: Epididymis adipose tissue removal
CIB: Cardiomyocyte isolation buffer
MEM: Minimum Essential Medium Eagle
BSA: Bovine serum albumin
EAT: Epididymis adipose tissue
TEM: Transmission electron microscopy
NTA: Nanoparticle tracking analysis
cTnT: Cardiac troponin T
UT: Untreated group
MMP: Mitochondrial membrane potential
DOX: Doxorubicin
mTORC2: Mammalian target of rapamycin complex 2
NC: Negative control
TAC: Transverse aortic constriction
