# Supplementary figures and images for: Secretion of miRNA-326-3p by senescent adipose exacerbates myocardial metabolism in diabetic mice
Source: J Transl Med. 2022 Jun 21;20:278. doi: 10.1186/s12967-022-03484-7 (PMC9210699; doi:10.1186/s12967-022-03484-7)

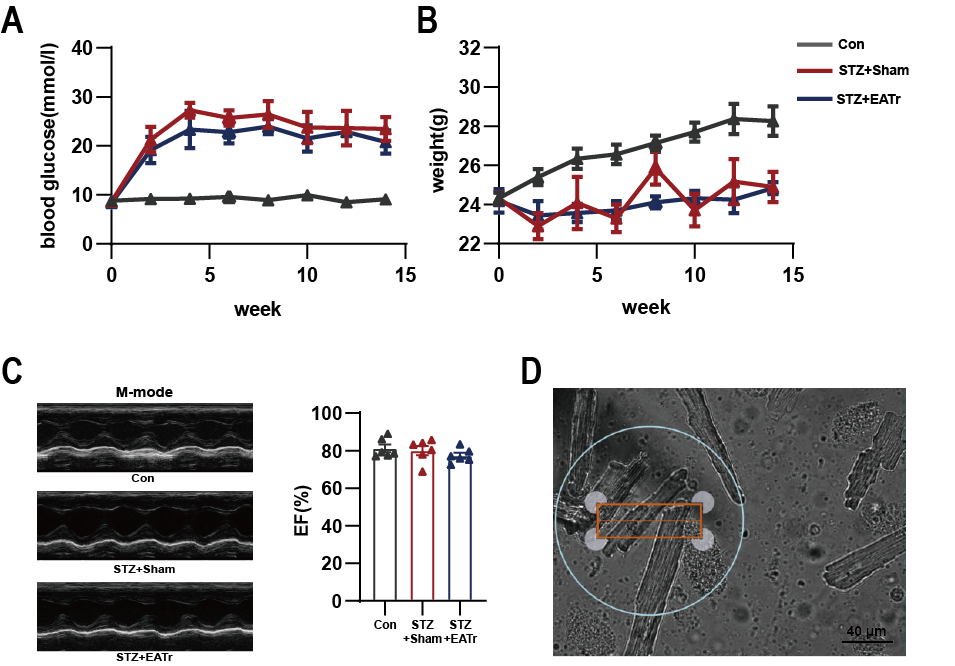

Supplement: Supplementary file 1 — Additional file 1: Figure S1. Blood glucose and weight monitoring of experimental mice. (A) Blood glucose tracking of mice after STZ injection. (B) Weight monitoring over time of mice after STZ injection. (C) Representative echocardiography M-model of long axis view used to evaluate ejection fraction (n = 6 per group). (D) Representative image of Langendorff-purified adult mouse ventricular myocytes (AMVMs). [file 12967_2022_3484_MOESM1_ESM.tif]

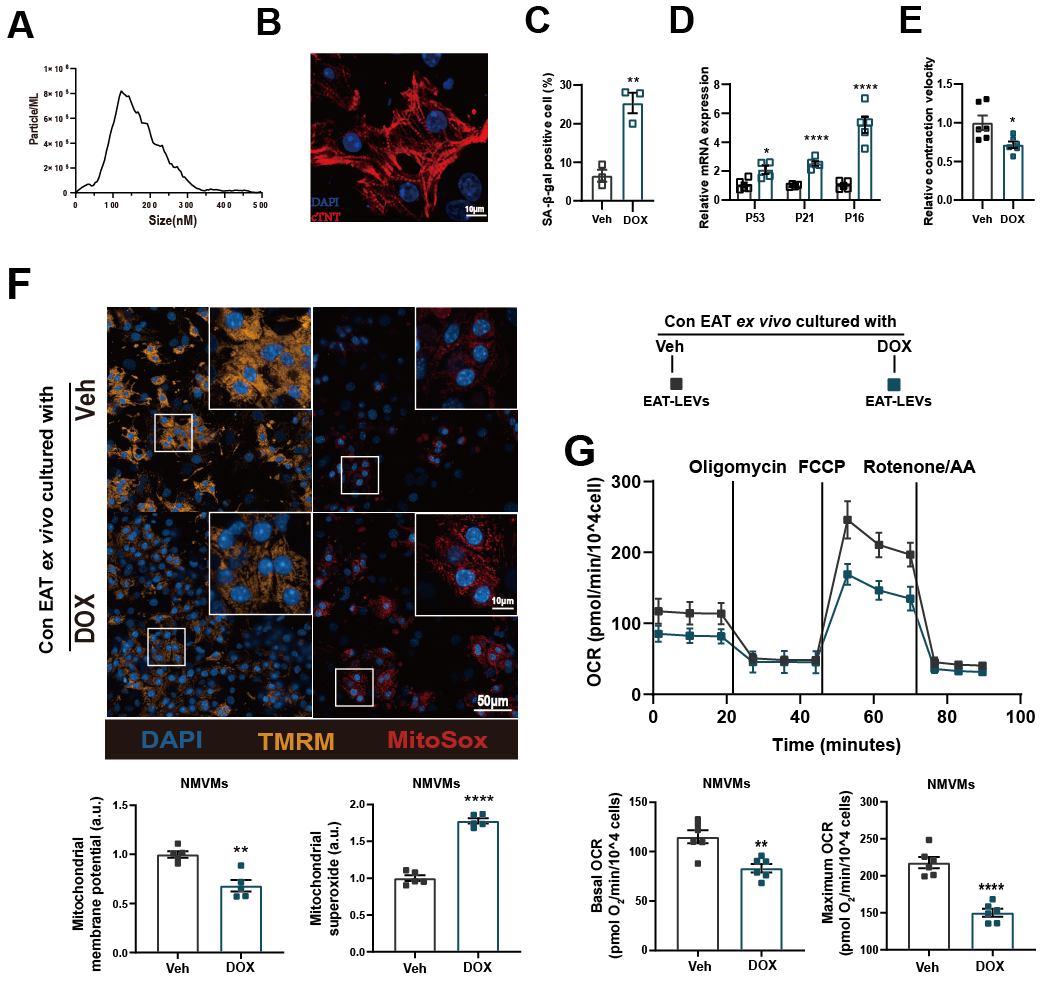

Supplement: Supplementary file 2 — Additional file 2: Figure S2. LEVs secreted by senescent EAT impair mitochondrial functions in NMVMs. (A) Distribution analysis of particle size for LEVs using nanosight tracking analysis. (B) Representative micrograph in which NMVMs were stained with cardiac troponin T and DAPI. (C) Detection of SA-β-gal activity in Con-EAT cultured ex vivo in presence of DOX or Veh (n = 3 per group). (D) Relative gene expression of age-associated genes in Con-EAT cultured ex vivo in presence of DOX or Veh (n = 3 per group). (E) Contractile velocity of NMVMs treated with LEVs from Con-EAT cultured ex vivo in presence of DOX or Veh (n = 6 per group). (F) Representative micrograph of NMVMs treated with LEVs from Con-EAT cultured ex vivo in presence of DOX or Veh and stained for mitochondrial membrane potential (TMRM) or mitochondrial superoxide (MitoSox) and DAPI, mitochondrial membrane potential and mitochondrial superoxide relative intensity of fluorescence are shown (n = 5 per group). (G) Real-time oxygen consumption rates (OCR) were evaluated for NMVMs treated with LEVs from Con-EAT cultured ex vivo in presence of DOX or Veh, basal and maximal respiration rates are shown (n = 8 per group). Data are presented as the mean ± SEM; *P < 0.05, **P < 0.01, ****P < 0.0001 compared to Veh group. [file 12967_2022_3484_MOESM2_ESM.tif]

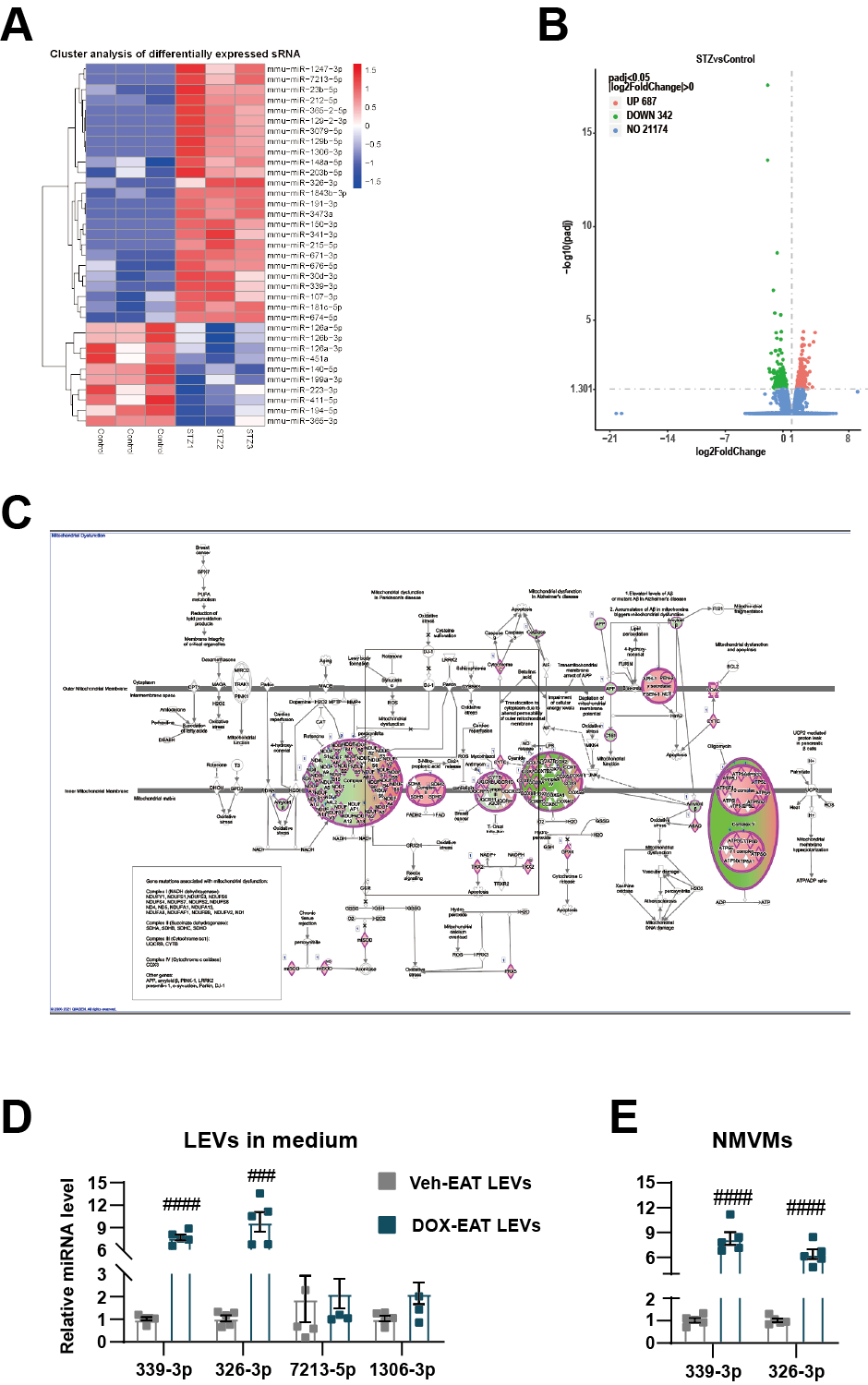

Supplement: Supplementary file 3 — Additional file 3: Figure S3. miRNA-seq of LEVs and mRNA-seq of AMVMs and RT-qPCR verification for DOX-EAT LEVs. (A) LEVs from Con-EAT and STZ-EAT were purified and subjected to non-coding RNA-Seq. Heat map of differentially expressed LEV-miRNAs derived form Con- or STZ-EAT are shown. (B) Volcano plot of differentially expressed genes from isolated AMVMs RNA-seq. (C) Diagram of mitochondria-related signaling pathways, green = up-regulated different genes, red = down-regulated genes. (D) Expression level of miRNA in Veh- or DOX-Con-EAT LEVs collected from adipose tissue conditioned medium (n = 5 per group). (E) Expression level of miRNA-339-3p and miRNA-326-3p in NMVMs treated with LEVs from Con-EAT cultured ex vivo in presence of DOX or Veh (n = 5 per groups). Data are presented as the mean ± SEM; ###P < 0.001, ####P < 0.0001 compared to Veh group. [file 12967_2022_3484_MOESM3_ESM.tif]

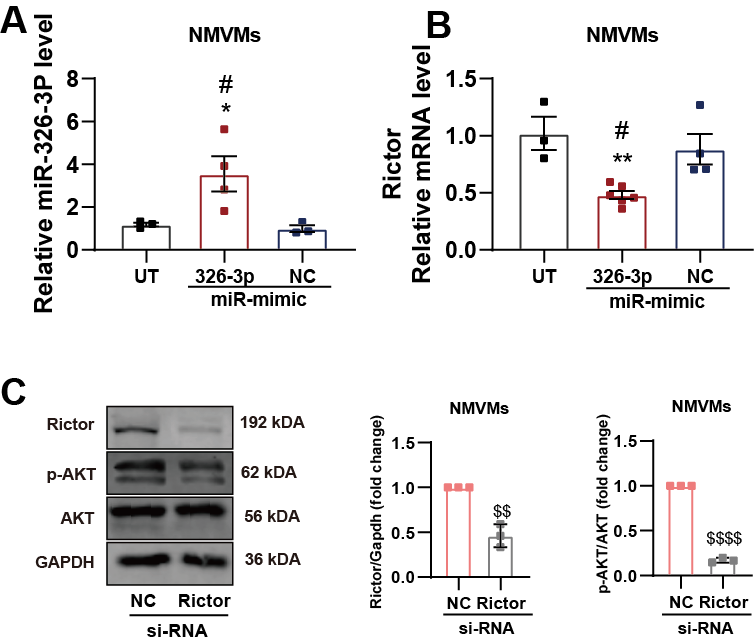

Supplement: Supplementary file 4 — Additional file 4: Figure S4. Characterization of miRNA-326-3p mimic transfection efficiency in NMVMs. (A) Relative miRNA-326-3p expression level in UT or NMVMs transfected with 326-3p- or NC-miR-mimic (n = 3 per group). (B) Relative Rictor mRNA expression level in UT or NMVMs transfected with 326-3p- or NC-miR-mimic (n = 3–6 per group). (C) Protein levels of Rictor, p-AKT, AKT and Gapdh in NMVMs transfected with Rictor- or NC-siRNA. (n = 3 per group). Data are presented as the mean ± SEM; *P < 0.05, **P < 0.01 compared to UT group; #P < 0.05 compared to NC-miR-mimic group; $$P < 0.01, $$$$P < 0.0001 compared to NC-siRNA group. [file 12967_2022_3484_MOESM4_ESM.tif]

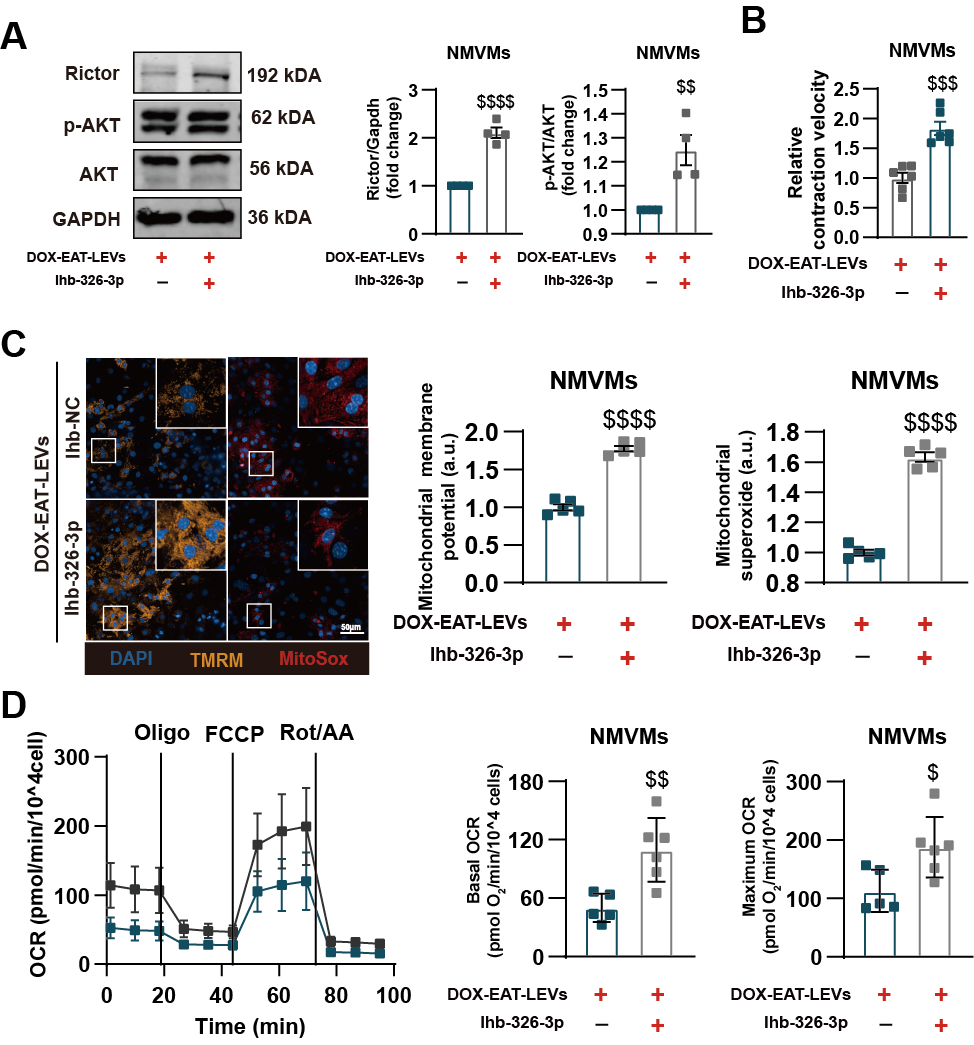

Supplement: Supplementary file 5 — Additional file 5: Figure S5. DOX-induced senescent EAT derived LEVs repress NMVMs Rictor expression via miRNA-326-3p. (A) Protein levels of Rictor, p-AKT, AKT and Gapdh in DOX-EAT LEVs co-cultured NMVMs transfected with miR decoy (Inh-326-3p) were evaluated by immunoblotting. (n = 3 per group). DOX-EAT LEVs co-cultured NMVMs were transfected with Inh-326-3p or untreated and (B) contractile velocity (n = 6 per group), (C) mitochondrial membrane potential (TMRM) and mitochondrial superoxide (MitoSox) (n = 5 per group), and (D) mitochondrial respiration (n = 6 per group) were assayed. Data are presented as the mean ± SEM; $P < 0.05, $$P < 0.01, $$$P < 0.001, $$$$P < 0.0001 compared to DOX-EAT LEVs group. [file 12967_2022_3484_MOESM5_ESM.tif]

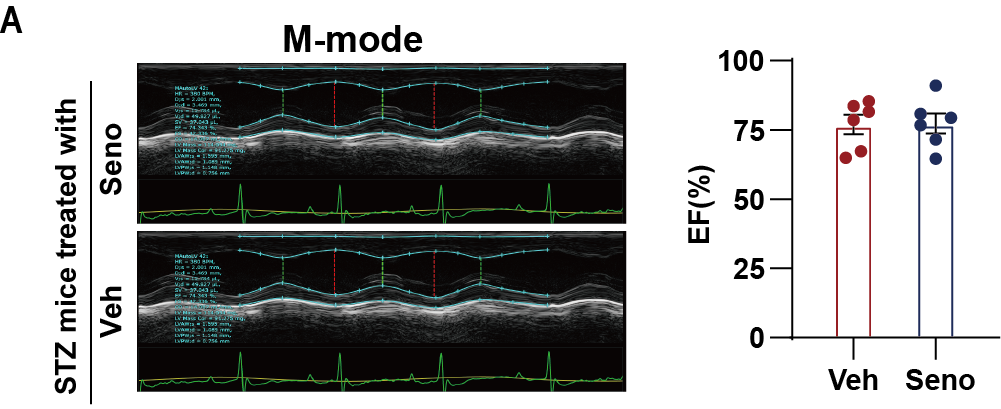

Supplement: Supplementary file 6 — Additional file 6: Figure S6. Preservation of cardiac systolic function in STZ mice treated with senolytic. (A) Representative M-model long axis echocardiography and ejection fraction (EF%) of Seno or Veh treated STZ mice (n = 6 per group). [file 12967_2022_3484_MOESM6_ESM.tif]
