# Supplementary material for: Secretion of miRNA-326-3p by senescent adipose exacerbates myocardial metabolism in diabetic mice
Source: J Transl Med. 2022 Jun 21;20:278. doi: 10.1186/s12967-022-03484-7 (PMC9210699; doi:10.1186/s12967-022-03484-7)
Supplement: Supplementary file 7 — Additional file 7: Table S1. Primers used in experiment [file 12967_2022_3484_MOESM7_ESM.docx]

| **Peimers** | | |
| --- | --- | --- |
| miDETECT A Track^TM^ cel-miR-39-3p Forward Primer | Ruibio | Cat#: miRA0000010 |
| miDETECT A Track^TM^ mmu-miR-7213-5p Forward Primer | Ruibio | Cat#: miRA1001143 |
| miDETECT A Track^TM^ mmu-miR-365-2-5p Forward Primer | Ruibio | Cat#: miRA100853 |
| miDETECT A Track^TM^ mmu-miR-107-3p Forward Primer | Ruibio | Cat#: miRA0000010 |
| miDETECT A Track^TM^ mmu-miR-1247-3p Forward Primer | Ruibio | Cat#: miRA1001717 |
| miDETECT A Track^TM^ mmu-miR-129-2-3p qPCR Forward Primer | Ruibio | Cat#: miRA1000240 |
| miDETECT A Track^TM^ mmu-miR-23b-5p Forward Primer | Ruibio | Cat#: miRA0000104 |
| miDETECT A Track^TM^ mmu-miR-326-3p Forward Primer | Ruibio | Cat#: miRA1000021 |
| miDETECT A Track^TM^ mmu-miR-671-3p Forward Primer | Ruibio | Cat#: miRA1000066 |
| miDETECT A Track^TM^ mmu-miR-339-3p Forward Primer | Ruibio | Cat#: miRA1000865 |
| miDETECT A Track^TM^ mmu-miR-1306-3p Forward Primer | Ruibio | Cat#: miRA101109 |
| miDETECT A Track^TM^ mmu-miR-3473a Forward Primer | Ruibio | Cat#: miRA101284 |
| miDETECT A Track^TM^ mmu-miR-212-5p Forward Primer | Ruibio | Cat#: miRA101108 |
| miDETECT A Track^TM^ mmu-miR-129b-5p Forward Primer | Ruibio | Cat#: miRA1000473 |
| miDETECT A Track^TM^ mmu-miR-148a-5p Forward Primer | Ruibio | Cat#: miRA1000353 |
| miDETECT A TrackTM mmu-miR-150-3p Forward Primers | Ruibio | Cat#: miRA1000917 |
| miDETECT A Track^TM^ mmu-miR-191-3p Forward Primer | Ruibio | Cat#: miRA100939 |
| miDETECT A Track^TM^ mmu-miR-674-5p Forward Primer | Ruibio | Cat#: miRA1000833 |
| Bulge-Loop mmu-miR-326-3p Primer Set | Ruibio | Cat#: miRA100976 |
| Bulge-Loop U6 qPCR Primer Set | Ruibio | Cat#: MQPS0000002 |
| Bulge-Loop cel-miR-39-3p Primer Set | Ruibio | Cat#: MQPS0000071 |
| Bulge-Loop hsa-miR-326 Primer Set | Ruibio | Cat#: MQPS0002718 |
| Bulge-Loop hsa-miR-339-3p Primer Set | Ruibio | Cat#: MQPS0001082 |
| Bulge-Loop mmu-miR-339-3p Primer Set | Ruibio | Cat#: MQPS0002732 |
| miDETECTTM cel-miR-39-3p Standard RNA | Ruibio | Cat#: MQPS0001061 |
| micrONTM mmu-miR-326-3p mimic | Ruibio | Cat#: miR10000559 |
| micrON mimic NC #22 | Ruibio | Cat#: miRB0000010 |
| micrOFFTM mmu-miR-326-3p inhibitor | Ruibio | Cat#: miR20000559 |
| micrOFF inhibitor NC #22 | Ruibio | Cat#: miR2N0000001 |
| Primer Gapdh  (forward):ATCATCCCTGCATCCACT  (reverse):ATCCACGACGGACACATT | Sangon | Custom synthesis |
| Primer p53  (forward):CGACCTATCCTTACCATCATCACA  (reverse):TTCTGTACGGCGGTCTCTC | Sangon | Custom synthesis |
| Primer p21  (forward):AACATCTCAGGGCCGAAA  (reverse):TGCGCTTGGAGTGATAGAAA | Ruibio | Cat#: miR1N0000001 |
| Primer p16  (forward):TGCTCAACTACGGTGCAGATTC  (reverse):ATGTCTTGATGTCCCCGCTCT | Sangon | Custom synthesis |
| Primer Rictor  (forward):CACTCGCATAGCCACCATCTACAC  (reverse):GTCCCAATCAGATTCCAATCCCAGTC | Sangon | Custom synthesis |
