# Supplementary material for: Secretion of miRNA-326-3p by senescent adipose exacerbates myocardial metabolism in diabetic mice
Source: J Transl Med. 2022 Jun 21;20:278. doi: 10.1186/s12967-022-03484-7 (PMC9210699; doi:10.1186/s12967-022-03484-7)
Supplement: Supplementary file 8 — Additional file 8: Table S2. Antibodies used in experiment. [file 12967_2022_3484_MOESM8_ESM.docx]

| **Antibodies** | | |
| --- | --- | --- |
| Rabbit monoclonal anti-p53 | Proteintech | Cat#: 60283-2-Ig; RRID: AB_2881401  (1:500 dilution) |
| Mouse monoclonal anti-p21 | Santa Cruz | Cat#: sc-6246, RRID: AB_628073  (1:50 dilution) |
| Mouse monoclonal anti-Rictor | Proteintech | Cat#: 66867-2-Ig (1:1000 dilution) |
| Rabbit monoclonal anti-AKT | Proteintech | Cat#:10176-2-AP (1:1000 dilution) |
| Rabbit monoclonal anti-Phospho-AKT(Ser473) | Proteintech | Cat#: 66444-1-Ig (1:1000 dilution) |
| Rabbit monoclonal anti-GAPDH | Proteintech | Cat#: 10494-1-AP (1:1000 dilution) |
| Anti-rabbit IgG (H+L) | CST | Cat#: 5151S |
| Anti-mouse IgG (H+L) | CST | Cat#: 5257S |
| Alexa Fluor 594 goat anti-rabbit IgG | Invitrogen | Cat#: A32742, RRID: AB_276282  (1:1000 dilution) |
| Alexa Fluor 488 goat anti-mouse IgG | Invitrogen | Cat#: A32723, RRID: AB_2633275  (1:1000 dilution) |
