# Supplementary material for: Secretion of miRNA-326-3p by senescent adipose exacerbates myocardial metabolism in diabetic mice
Source: J Transl Med. 2022 Jun 21;20:278. doi: 10.1186/s12967-022-03484-7 (PMC9210699; doi:10.1186/s12967-022-03484-7)
Supplement: Supplementary file 9 — Additional file 9: Table S3. Reagent or resource used in experiment. [file 12967_2022_3484_MOESM9_ESM.docx]

| **REAGENT or RESOURCE** | **SOURCE** | **IDENTIFIER** |
| --- | --- | --- |
| **Chemicals, Peptides, and Recombinant Proteins** | | |
| Streptozocin | Sigma-Aldrich | Cat#: S0130 |
| Dasatinib | MCE | Cat#: HY-10181 |
| Quercetin | Sigma-Aldrich | Cat#: Q4951 |
| Image-iT™ TMRM | Invitrogen | Cat#: I34361 |
| MitoSOX™ Red | Invitrogen | Cat#: M36008 |
| TRIzol | Invitrogen | Cat#: 15596018 |
| Dr.GenTLE™ Precipitation Carrier | TAKARA | Cat#: 9094 |
| Phalloidin-Alexa Fluor 594 | Beyotime | Cat#: C2205S |
| laminin | Invitrogen | Cat#: L2020 |
| Doxorubicin | MCE | Cat#: HY-15142 |
| Critical Commercial Assays | | |
| The PKH67 Green Fluorescent Cell Linker Mini Kit | Sigma-Aldrich | Cat#: MINI67 |
| Seahorse XF Cell Mito Stress Test Kit | Agilent | Cat#:103010-100 |
| β-Galactosidase Staining Kit | Solarbio | Cat#: G1580 |
| TransZol Up Plus RNA Kit | TransGen | Cat#: ER501-01 |
| HiScriptⅡ Reverse Transcriptase kit | Vazyme | Cat#: R201-01/02 |
| miDETECT A Track miRNA RT-qPCR Starter Kit | Ruibo | Cat#: C10712-1 |
| **Deposited Data** | | |
| RNA-seq data | this paper |  |
| **Experimental Models: Organisms/Strains** | | |
| Mouse C57BL/6J | GemPharmatech Co. | N/A |
| **Software and Algorithms** | | |
| CytoMotion | IonOptix | https://www.ionoptix.com/ |
| Vevo Lab | FUJIFILM | https://www.visualsonics.com |
| ZEN | ZEISS | https://www.zeiss.com.cn/microscopy/products/microscope-software/zen.html |
| IX83 software (IX3-BSW) | Olympus | https://www.olympus-lifescience.com.cn/zh/support/downloads/#!dlOpen=%23detail847252279 |
| Seahorse Wave | Agilent | https://www.agilent.com.cn/zh-cn/product/cell-analysis/real-time-cell-metabolic-analysis/xf-software/seahorse-wave-desktop-software-740897 |
| Matlab | Mathworks | https://www.mathworks.com/ |
| Image j | National Institutes of Health | https://imagej.nih.gov/ij/ |
| GraphPad Prism 8 | GraphPad Software | www.graphpad.com |
